# Immune modulation resulting from MR-guided high intensity focused ultrasound in a model of murine breast cancer

**DOI:** 10.1038/s41598-020-80135-1

**Published:** 2021-01-13

**Authors:** Brett Z. Fite, James Wang, Aris J. Kare, Asaf Ilovitsh, Michael Chavez, Tali Ilovitsh, Nisi Zhang, Weiyu Chen, Elise Robinson, Hua Zhang, Azadeh Kheirolomoom, Matthew T. Silvestrini, Elizabeth S. Ingham, Lisa M. Mahakian, Sarah M. Tam, Ryan R. Davis, Clifford G. Tepper, Alexander D. Borowsky, Katherine W. Ferrara

**Affiliations:** 1 Department of Radiology, Stanford University, 3165 Porter Dr, Palo Alto, CA 94305 USA; 2 Department of Biomedical Engineering, Stanford University, Palo Alto, CA 94305 USA; 3 Department of Biomedical Engineering, University of California Davis, Davis, CA 95616 USA; 4 Department of Pathology and Laboratory Medicine, School of Medicine, University of California Davis, Sacramento, CA 95817 USA; 5 Department of Biochemistry and Molecular Medicine, School of Medicine, University of California Davis, Sacramento, CA 95817 USA

**Keywords:** Preclinical research, Biomedical engineering

## Abstract

High intensity focused ultrasound (HIFU) rapidly and non-invasively destroys tumor tissue. Here, we sought to assess the immunomodulatory effects of MR-guided HIFU and its combination with the innate immune agonist CpG and checkpoint inhibitor anti-PD-1. Mice with multi-focal breast cancer underwent ablation with a parameter set designed to achieve mechanical disruption with minimal thermal dose or a protocol in which tumor temperature reached 65 °C. Mice received either HIFU alone or were primed with the toll-like receptor 9 agonist CpG and the checkpoint modulator anti-PD-1. Both mechanical HIFU and thermal ablation induced a potent inflammatory response with increased expression of *Nlrp3*, *Jun, Mefv, Il6* and *Il1β* and alterations in macrophage polarization compared to control. Furthermore, HIFU upregulated multiple innate immune receptors and immune pathways, including *Nod1, Nlrp3, Aim2, Ctsb, Tlr1/2/4/7/8/9, Oas2, and RhoA*. The inflammatory response was largely sterile and consistent with wound-healing. Priming with CpG attenuated *Il6* and *Nlrp3* expression, further upregulated expression of *Nod2*, *Oas2, RhoA, Pycard, Tlr1/2 and Il12,* and enhanced T-cell number and activation while polarizing macrophages to an anti-tumor phenotype. The tumor-specific antigen, cytokines and cell debris liberated by HIFU enhance response to innate immune agonists.

## Introduction

High intensity focused ultrasound (HIFU) is a non-invasive method for the treatment of solid tumors, including those grown in pancreas, liver, kidney, and prostate^1–4^. HIFU is capable of delivering intense acoustic energy to a small focus with high spatial precision, preventing damage to surrounding tissue while resulting in the rapid destruction of tissue within the focal volume through mechanical disruption, thermal damage, or a combination of the two. Furthermore, magnetic resonance guided focused ultrasound (MRgFUS) affords the ability to non-invasively measure temperature in real time, facilitating precise control of temperature and thermal dose^5–8^.

HIFU ablation can exert both thermal and mechanical effects on the targeted tissue. Thermal effects are produced when tissue absorbs acoustic energy, resulting in an increase in temperature; the treatment time and acoustic intensity can be varied to achieve a wide variety of temperature elevations, from only 1–2 °C up to the boiling point of tissue. Mechanical effects primarily result from the production of cavitation bubbles (histotripsy) or microscopic boiling bubbles (boiling histotripsy)^9,10^. Mechanical disruption typically results in homogenization and eventual resorption of the liquefied tissue with a narrow transition region^11,12^. Conversely, thermal ablation has a broader transition region due to heat diffusion and results in the formation of a scar, which is not as easily resorbed and is resistant to reinvasion. Both mechanical and thermal HIFU rapidly and effectively destroy tissue and have been employed for local control of tumors.

Pre-clinical results suggest local ablative techniques can cause immunogenic cell death (ICD), increased tumor infiltration of macrophages, CD4^+^ and CD8^+^ lymphocytes^13^, release of endogenous danger signals^14^ and tumor specific antigens^15^, and can result in an antitumor response^16–19^. Clinical results also demonstrate modulation of the immune system following HIFU^13,20^. However, ablation alone generates only a weak abscopal effect^16,21^; the initial inflammatory response, mediated by HIFU’s release of damage associated molecular patterns (DAMPs) from damaged or destroyed cells is not accompanied by simultaneous pathogen associated molecular pattern (PAMP) recognition, which is likely one major requirement for generating a strong immune response^22^. Thus, without additional stimuli to innate immune receptors^23^, HIFU alone is insufficient to overcome tumor-mediated immune suppression in the vast majority of cases. Nevertheless, the cytokines and cell debris liberated by HIFU can be harnessed for immune stimulation when combined with immunotherapies.

Previously, we have combined HIFU with checkpoint modulation (anti-PD-1) and toll-like receptor (TLR) 9 ligation (using CpG) to generate a durable cure in a majority of mice^15,21^ in a “priming” strategy. Our work has demonstrated the importance of timing and sequence of multiple immunomodulatory therapeutics^24–26^. Aside from debulking, HIFU’s ability to release tumor-specific antigen and its effect on the tumor immune environment can synergize with immunotherapeutic agents. However, the dynamism of the tumor cytokine and immune cell population profile in response to immunotherapeutics and HIFU necessitates further investigation to optimize their combination. Furthermore, the variability in tissue effects generated through different HIFU treatment schemes (e.g. thermal ablation, hyperthermia, histotripsy, boiling histotripsy, low intensity ultrasound) may necessitate different therapeutic combinations to achieve optimal therapeutic effects. Herein, we aim to elucidate the immune effects of HIFU and its combination with immunotherapy. Specifically, we use a combination of anti-PD-1 therapy to increase T-cell response and the TLR9 agonist CPG to activate the myeloid compartment and increase T-cell infiltration within the tumor. Without a robust T-cell infiltrate in the tumor, PD-1 treatment alone may prove less efficacious. We first start with dimensional reduction analysis of transcriptomes from various treatments to understand the differences across treatment combinations. We then demonstrate that while all therapies reduce tumor burden and downregulate cancer and proliferation-related genes, HIFU induces a potent inflammatory response, which is mitigated by priming with an innate immune agonist. Further, thermal ablation upregulates innate immune receptors, and this effect is enhanced by addition of an agonist. Activation of the innate immune system, especially in concert with agonists, generates a systemic, adaptive response, mediated through T cells, B220^+^ cells, and cytokines.

## Results

### Overall summary of the magnitude of the effect

We hypothesized that different treatment protocols would elicit distinct biological effects observable from transcriptomic expression data. Our choice of time points was based on the ultrasound parameters we chose to employ and our experience with their biological effects and immunohistochemistry studies; namely, we found cell death/destruction was significantly more rapid with mechanical ablation than with thermal ablation. Previously reported results with histotripsy^27^ have also suggested cell death immediately following treatment. Here, matching immunohistochemistry (IHC) datasets at 72 h and 7 days and flow cytometry at 72 h were acquired for both treatments. These datasets were augmented by an earlier time point for mechanical ablation (24 h for transcriptomics and 48 h for IHC) and a later dataset for thermal ablation (7 day transcriptomics).

**Figure 1:**
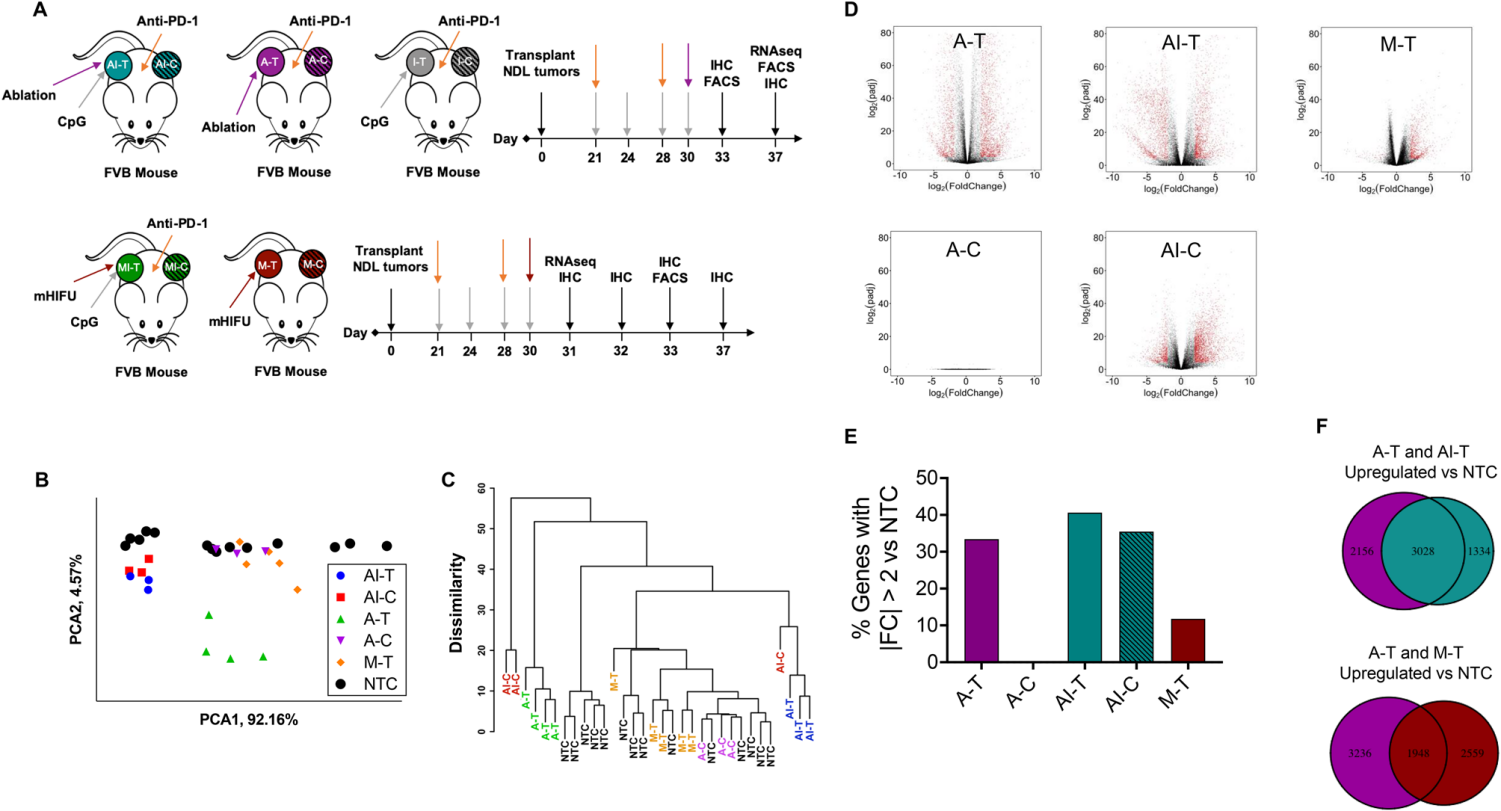
HIFU reduces viable tumor and downregulates cancer and proliferation related genes and pathways. In all cases, “-T” indicates the directly-treated tumor and “-C” indicates the distant/contralateral tumor. (**A**) Schematic illustrating each treatment cohort and timing of treatments and endpoints for animals treated with (top) thermal ablation and (bottom) mechanical HIFU. (**B**) Principal component analysis biplot of the first two components of treatment cohorts. (**C**) Hierarchical clustering dendrogram of the entire transcriptome for each individual tumor within each treatment cohort. (**D**) Volcano plots of each treatment cohort plotting log_2_ fold change versus negative log_2_ adjusted p-value of each gene’s expression compared to control, plotted only for genes whose expression is significantly changed (p < 0.05). Red points indicate genes where the absolute value of the log_2_ fold change is greater than 2. (**E**) Bar plot summarizing the percentage of genes with expression |FC|> 2 out of all significantly altered genes for each cohort compared to control. (**F**) Venn diagrams illustrating overlap between thermal ablation and thermal ablation with immunotherapy and between thermal ablation and mechanical HIFU of upregulated genes (compared to control). Volcano plots, Venn diagrams, and the hierarchical clustering dendrogram were created in R Studio v1.2.5001 (https://rstudio.com).

To test this hypothesis, we first performed principal component analysis on whole transcriptomes from our NDL tumor-bearing mouse cohorts and found that different treatments (Fig. 1A) have distinct clustering patterns. Both the directly-treated (AI-T) and distant (AI-C) tumors of animals that were treated with immunotherapy and thermal ablation clustered together (Fig. 1B). Tumors treated with thermal ablation alone (A-T) clustered together and were distinct from control tumors and tumors from animals that received immunotherapy prior to thermal ablation. AI-T tumors were 2.2 times further from control tumors than A-T tumors (calculated as the mean Euclidean distance between each cohort and control across all principal components), and AI-C tumors were 5.1 times further from control tumors than A-C tumors. Tumors treated with mechanical HIFU (M-T) and distant tumors in animals that received thermal ablation alone (A-C) clustered with control tumors (NTC), consistent with the lack of response to therapy in these cohorts. A hierarchical clustering of the entire transcriptome (Fig. 1C) similarly demonstrated separate clustering of AI-T, AI-C, and A-T, while M-T, A-C, and NTC tumors clustered together.

To further understand the differences in gene expression across treatment cohorts, we utilized volcano plots of log2 fold change of gene expression (compared to control) with respect to the negative log2 of the adjusted p value of the change in expression (Fig. 1D) to illustrate the differences between the treatment cohorts. In the absence of immunotherapy, gene expression in treated tumors was altered with both mechanical (4673 genes upregulated versus 4011 genes downregulated) and thermal (6406 genes upregulated versus 5553 genes downregulated) ablation. Changes in distant tumors were small; A-C tumors exhibit no change from control (one gene had p_adj_ < 0.05) (Fig. 1B), and a similar result was observed with mechanical HIFU. After priming with immunotherapy prior to ablation, similar numbers of genes were downregulated and upregulated as compared to HIFU alone (4560 genes upregulated and 4755 genes downregulated for AI-T) in the treated tumor. However, the magnitude of the effect was greater (Fig. 1E) in AI-T tumors; 41% of AI-T genes were differentially expressed with |FC|> 2 (compared to control) as compared to 33% for A-T and 11.8% for M-T. In distant tumors treated with the combined protocol, 5152 genes were upregulated and 4396 downregulated compared to a single gene in A-C tumors. Specific genes associated with adaptive immune response that were upregulated in AI-T compared to control and A-T included *Prf1*, *Gzmb*, *Batf3*, *Pdcd1*, *Ifng*, *Cd244a*, *Tigit*. A greater fraction of upregulated genes overlap between A-T and AI-T than between A-T and M-T (Fig. 1F) suggesting mechanistic differences between thermal ablation and mechanical HIFU therapy.

### HIFU reduces viable tumor

**Figure 2:**
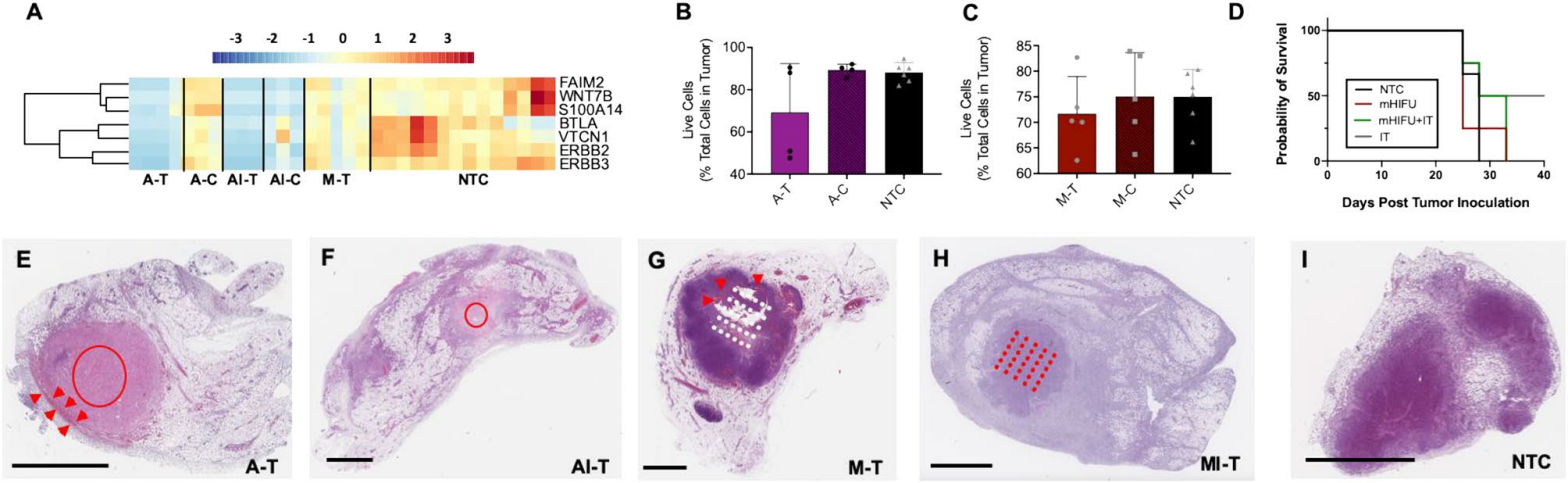
HIFU reduces viable tumor and downregulates cancer and proliferation related genes and pathways. (**A**) Heatmap visualization of Z-scores of cancer and proliferation-related genes for each treatment cohort. Live cells as measured with flow cytometry of the (**B**) thermal ablation and (**C**) mechanical ablation cohorts. (**D**) Survival proportions of control animals compared to animals that were treated with mechanical HIFU alone, immunotherapy alone, or the combination of mechanical HIFU and immunotherapy. H&E sections of (**E**) a thermally-ablated tumor (arrows indicate rim of viable tumor cells), (**F**) a tumor that received both thermal ablation and immunotherapy, (**G**) a tumor treated with mechanical HIFU (arrows indicate hemorrhage), (**H**) a tumor that received both mechanical HIFU and immunotherapy, and (**I**) an untreated control tumor. Scale bar represents 2 mm in all cases. Time points for RNA-seq are: 1 week post ablation for all cohorts that received thermal ablation, and 24 h post HIFU for cohorts that received mechanical HIFU. For flow cytometry studies, time points are 72 h for cohorts that received mechanical HIFU and 1 week for cohorts that received thermal ablation alone. Time points for IHC are 72 h post ablation for thermal ablation alone, 24 h for mechanical HIFU alone, and 1 week for both thermal and mechanical ablation plus immunotherapy. Red and white overlays on histological sections indicate the insonation pattern performed (either circular continuous wave for thermal ablation or a grid pattern for mechanical HIFU). The heatmap was created in R Studio v1.2.5001 (https://rstudio.com).

Since HIFU is a non-invasive method for tumor debulking, we sought to characterize the cellular and biomolecular differences of thermal ablation, mechanical HIFU, and thermal ablation combined with immunotherapy. Cancer and proliferation-related genes such as *Wnt7b*, *S100a14*, and *Erbb2*, were downregulated by thermal ablation in directly-treated tumors (A-T) 1 week after ablation, and to a lesser extent, 24 h after mechanical HIFU (M-T) compared to control (NTC) (Fig. 2A). However, this effect did not extend to distant tumors (A-C), consistent with HIFU being a local ablative therapy. Addition of immunotherapy into the ablation protocol further reduced expression of proliferative and cancer-related genes in directly-treated (AI-T) tumors and distant (AI-C) tumors, demonstrating an abscopal effect. We have previously shown that such a combined protocol more than doubles survival in animals with multi-focal disease compared to immunotherapy alone^21^. Similarly, both thermal ablation (Fig. 2B) and mechanical HIFU (Fig. 2C) reduced viable tumor cells only in directly-treated, but not in distant, tumors. Both mechanical HIFU alone and mechanical HIFU pretreated with immunotherapy resulted in increased survival (Fig. 2D) compared to control, but were less efficacious than immunotherapy alone (IT). H&E stained sections of thermally-ablated tumors (Fig. 2E) exhibited a small rim of viable tumor cells while the combination of thermal ablation and immunotherapy eliminated the majority of viable tumor cells (Fig. 2F) and reduced tumor size. Mechanical HIFU caused substantial hemorrhage (Fig. 2G) but remaining viable tumor volume was larger compared to thermally-ablated tumors. However, the addition of immunotherapy substantially reduced viable tumor (Fig. 2H) compared to control tumors (Fig. 2I). Furthermore, the addition of immunotherapy to mechanical ablation reduced viable tumor at 24 h, 72 h, and 7 days post HIFU (Supplementary Fig. S2).

### HIFU induces a potent inflammatory response

**Figure 3:**
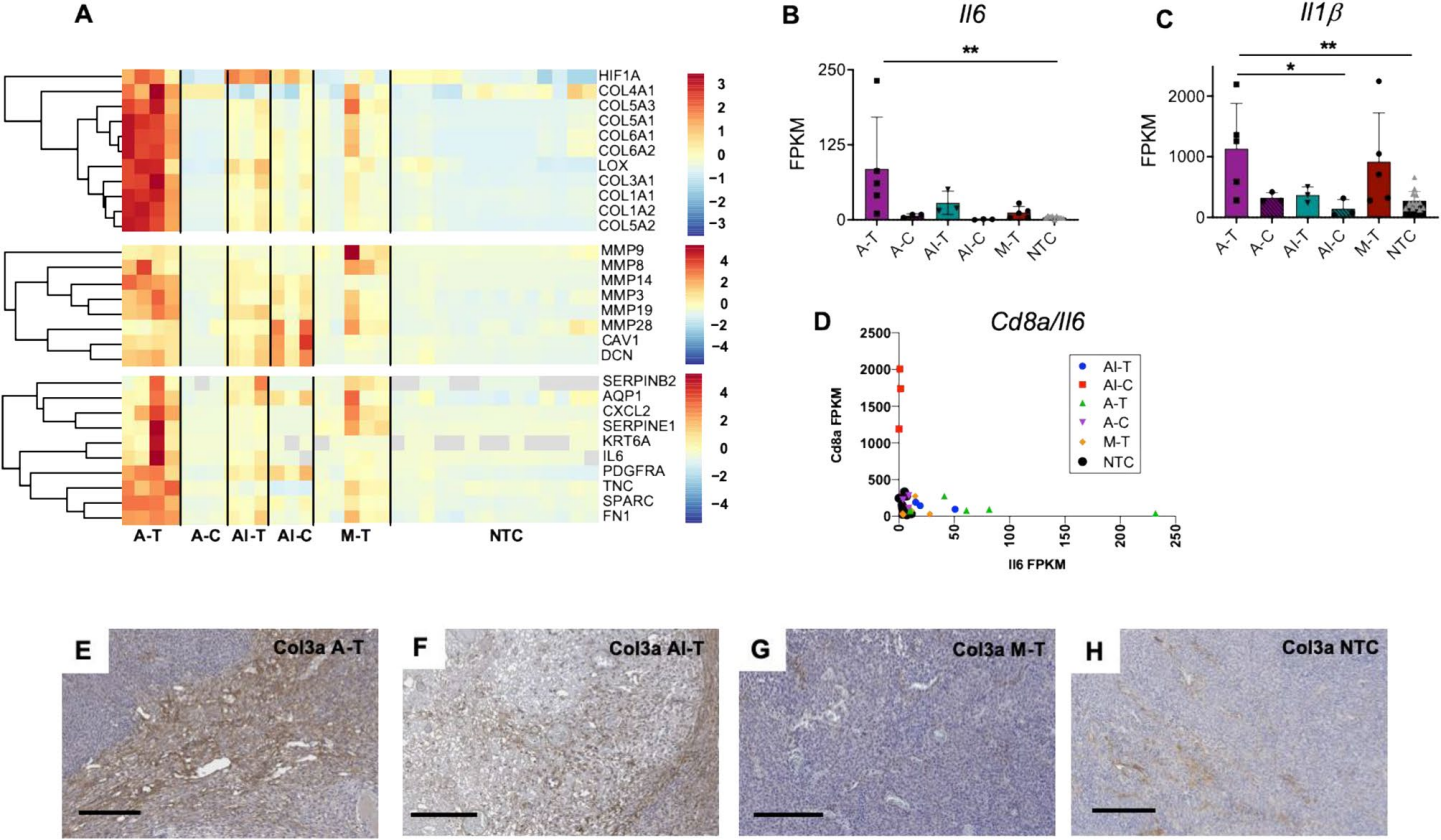
HIFU induces strong inflammatory response. (**A**) Z-scores of ECM remodeling related (top two panes) and wound healing related (bottom pane) genes for each treatment cohort. FPKM values for each cohort for (**B**) the inflammatory cytokine IL6, (**C**) an upstream regulator of inflammation IL1b. Scatter plot of the FPKM value ratios of (**D**) *Cd8*, versus *Il6* for each cohort. Col3a IHC stained sections of (**E**) a thermally-ablated tumor, (**F**) a tumor that received both thermal ablation and immunotherapy, (**G**) a tumor treated with mechanical HIFU, and (**H**) an untreated control tumor. Scale bar represents 200 µm in all cases. Time points are 1-week post ablation for all cohorts that received thermal ablation, and 24 h post HIFU for cohorts that received mechanical HIFU. Data are expressed as mean ± SD. *p < 0.05, **p < 0.01 (ordinary one-way ANOVA with Tukey correction for multiple hypotheses). The heatmap was created in R Studio v1.2.5001 (https://rstudio.com).

To understand the potential biological implications of HIFU induced tumor debulking, we performed gene ontology analysis with DAVID. Extracellular matrix (ECM) remodeling and wound healing pathways were induced by thermal ablation and mechanical HIFU (Fig. 3A), but the effect was greatest in thermally-ablated tumors, in which 165 cell adhesion related genes and 100 angiogenesis related genes were upregulated (Supplementary Table S1) compared to 64 and 45 genes, respectively for mechanical ablation.

We further reasoned that wound healing events would be correlated to an inflammatory response and should result in an up-regulation of pro-inflammatory genetic expression. Pro-inflammatory cytokines *Il6* (Fig. 3B) and *Il1β* (Fig. 3C) had increased expression in thermally-ablated tumors, while mechanical HIFU primarily increased expression of the upstream *Il1β*. Nevertheless, both mechanical HIFU and thermal ablation cohorts were enriched in the Il6 protein production pathway (GO:0032755) with 13 and 22 genes enhanced, respectively. Addition of immunotherapy prior to ablation decreased *Il6* gene expression as compared to thermal ablation alone, but remained higher than no treatment control *Il6* expression levels. Furthermore, for the combined protocol, the magnitude of the increases in expression of genes associated with the adaptive immune response was greater than that of *Il6* expression. In the immunotherapy-ablation cohort, distant tumors (AI-C) had a *Cd8a*:*Il6* expression ratio of 1708 (Fig. 3D) with *Il6* expression significantly attenuated. In addition, the immunotherapy-ablation protocol enriched the negative regulatory Il6 pathway (GO:0032715).

Type 3 collagen (Col3) is found in connective tissues, and upregulation of this type of collagen occurs during wound healing processes^28^. Furthermore, Col3A expression may “play a key role promoting myofibroblast proliferation and progression of fibroepithelial lesions in the breast”^29^. Thermal ablation increased Col3a protein within the treated tumor (Fig. 3E); however, priming with immunotherapy prior to thermal ablation reduced tumoral Col3a deposition (Fig. 3F). Mechanical ablation (Fig. 3G) did not alter Col3a levels as compared to untreated control tumors, with sparse, scattered Col3a observed throughout the tumor on IHC (Fig. 3H).

### Innate immune receptors and signaling pathways are locally upregulated by HIFU and systemically upregulated by adding immunotherapy

Activation of innate immune receptors, especially on myeloid cells, is a key element facilitating a systemic immune response after focal therapy. Pattern recognition receptors (PRRs) are innate immune receptors which recognize endogenous (DAMPs) or exogenous (PAMPs) molecular patterns associated with tissue damage and pathogen infection^30^. DAMPs, such as heat-shock proteins and HMGB1, are released following tissue damage. In the absence of additional stimuli, DAMP detection triggers a sterile inflammatory response that promotes wound healing and tissue repair. Detection of PAMPs, such as lipopolysaccharides and bacterial DNA, polarize the inflammatory response towards pathogen elimination. Innate immune PAMP sensors must be activated to achieve an anti-tumor immune response.

**Figure 4:**
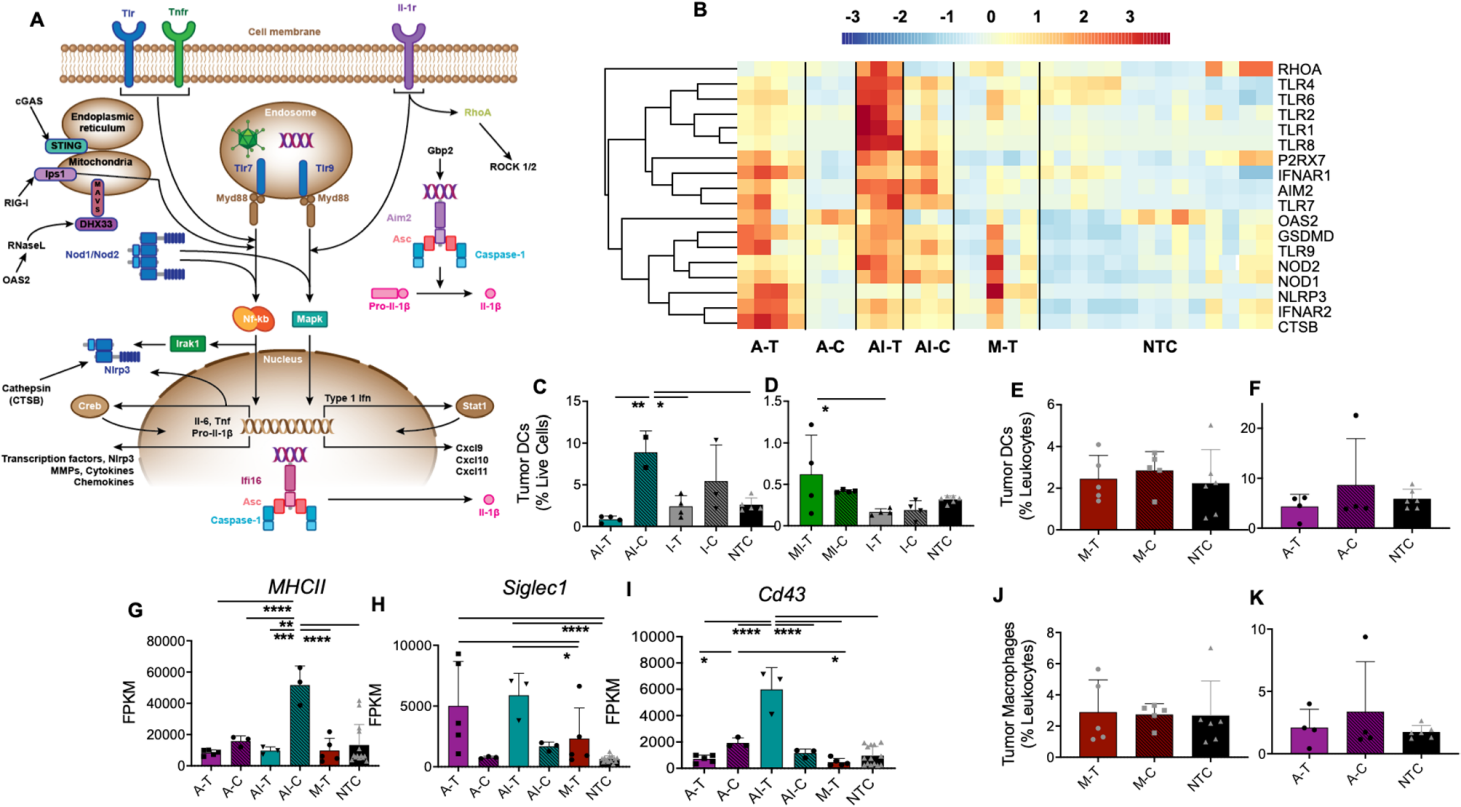
HIFU stimulates innate immune sensors and generates an innate response when combined with immunotherapy. (**A**) Schematic of innate immune receptors and signaling. (**B**) Heatmap visualization of Z-scores of innate immune sensors. Dendritic cells quantified with flow cytometry of the (**C**) thermal ablation pre-treated with immunotherapy, (**D**) mechanical HIFU pre-treated with immunotherapy, (**E**) mechanical HIFU, and (**F**) thermal ablation cohorts. FPKM values for each cohort for (**G**) *MHCII*, (**H**) *Siglec1*, and (**I**) *Cd43*. Macrophages as measured with flow cytometry of the (**J**) mechanical HIFU and (**K**) thermal ablation cohorts. Time points for RNA-seq are 1-week post ablation for all cohorts that received thermal ablation, and 24 h post HIFU for cohorts that received mechanical HIFU. For flow cytometry studies, time points are 72 h for cohorts that received mechanical HIFU or immunotherapy in addition to thermal ablation and 1 week for cohorts that received thermal ablation alone. Data are expressed as mean ± SD. *p < 0.05, **p < 0.01, ***p < 0.001, ****p < 0.0001 (ordinary one-way ANOVA with Tukey correction for multiple hypotheses). The heatmap was created in R Studio v1.2.5001 (https://rstudio.com).

Ablation alone releases DAMPs and upregulates multiple families of innate receptors, including Toll-like receptors (TLRs), NOD-like receptors (NLRs), cytosolic DNA sensors^30–32^ (Supplementary Fig. S3), and RIG-I-like receptors (RLRs) (Fig. 4A), which sensitize the innate compartment to detection of PAMPs. Specifically, expression of the innate sensors *Nod1, Nlrp3, Aim2, Ctsb, Tlr1/2/4/7/8/9, Oas2, and RhoA* increased following thermal ablation (Fig. 4B). However, thermal ablation also upregulates sterile inflammatory pathway genes including, *Nlrp3*, *Jun, Mefv, and Il6. Il1β* expression is enhanced with ablation and is associated with Th1 based cellular immunity via the caspase-1 pathway, Il1α can act as a DAMP and stimulate TLR pathways^33^. Notably, ablation alone did not alter innate immune expression profiles of distant tumors, which remained similar to control tumors.

The combined ablation-immunotherapy protocol produces a broad effect that is not limited to a particular receptor or cell-type; it upregulates innate receptors across multiple families and alters the polarization of infiltrating macrophages, thereby facilitating a strong systemic immune response. Addition of the innate agonist CpG to the ablation protocol further upregulates *Nod2*, *Oas2, RhoA, Pycard, Tlr1/2 and Il12* compared to ablation alone, augmenting the innate immune response and further enriching the following KEGG^34,35^ (developed by Kanehisa Laboratories) innate immune sensing and signaling pathways: NLR (KEGG: mmu04621), RIG-I (KEGG: mmu04622), cGAS cytosolic DNA sensing (KEGG: mmu04623), and TLR (KEGG: mmu04620). Innate immune response (GO:0045087) was the second most enriched pathway for AI-T, with expression of 111 genes altered, while response to lipopolysaccharide (LPS) (GO:0032496) was the second most enriched for both thermal and mechanical ablation, including 70 and 32 genes, respectively (Supplementary Table S2). Moreover, the combined immunotherapy-ablation protocol did not increase expression of *Nlrp3* and other inflammation-associated genes. Additionally, ligation of TLR9 by CpG potentiates macrophage phagocytosis^36^ and may synergize with ablation-induced DAMP release. For example, increased *Adgre1* expression in AI-T tumors is consistent with enhanced macrophage infiltration into the treated tumors.

To investigate the cellular effects of ablation, with or without immunotherapy, we homogenized tumor tissues and analyzed the cellular components via flow cytometry. Both thermal (Fig. 4C) and mechanical (Fig. 4D) HIFU increased dendritic cell (DC) infiltration when added to an immunotherapy protocol. However, infiltration of DCs was not significantly altered in tumors treated with either mechanical HIFU (Fig. 4E) or thermal ablation (Fig. 4F) in the absence of immunotherapy. Although we expect increased infiltration of antigen presenting cells following an ablative treatment, it is possible our choices of time points for flow cytometry did not capture the infiltration. Expression of *MHCII* was consistent with increased DC infiltration at distant sites in animals that received both thermal ablation and immunotherapy (Fig. 4G).

At the 1-week timepoint, although macrophage numbers (as assessed by flow cytometry) were similar across treatments, the phenotype was distinct. Siglec1 is expressed on macrophages and DCs associated with the presentation of dead cell antigens. HIFU upregulated *Siglec1* (Cd169) expression within directly-treated tumors, with AI-T > A-T > M-T (Fig. 4H). Expression of *Cd43*, the T-cell counterreceptor for Siglec1, increased only with the combined protocol as compared to control (Fig. 4I). Tumor macrophage infiltration was unchanged following either mechanical HIFU (Fig. 4J) or thermal ablation (Fig. 4K) as quantified by flow cytometry. Macrophage polarization was altered by both immunotherapy and mechanical HIFU, with distinct populations developing from 24 h to 7 days post HIFU (Supplemental Fig. S4). Macrophage polarization shifted towards M2 in tumors treated with mechanical HIFU (Supplementary Fig. S5A). With the addition of immunotherapy to thermal ablation, M2 macrophages were reduced compared to control or immunotherapy alone. Furthermore, the combined mechanical HIFU-immunotherapy protocol altered macrophage polarization to predominantly non-M1/M2, compared to control or mechanical HIFU alone (Supplementary Fig. S5B). IHC sections stained for F4/80 were concordant with flow cytometry results: macrophage infiltration was sparse for thermally-ablated tumors at the 1-week time point (Supplementary Fig. S5C); however, addition of immunotherapy increased macrophage recruitment and infiltration (Supplementary Fig. S5D). Mechanical HIFU did not alter macrophage infiltration (Supplementary Fig. S5E), and neither form of ablation was sufficient to enhance macrophage populations compared to control tumors (Supplementary Fig. S5F).

### Adaptive immune response is augmented by the addition of immunotherapy

**Figure 5:**
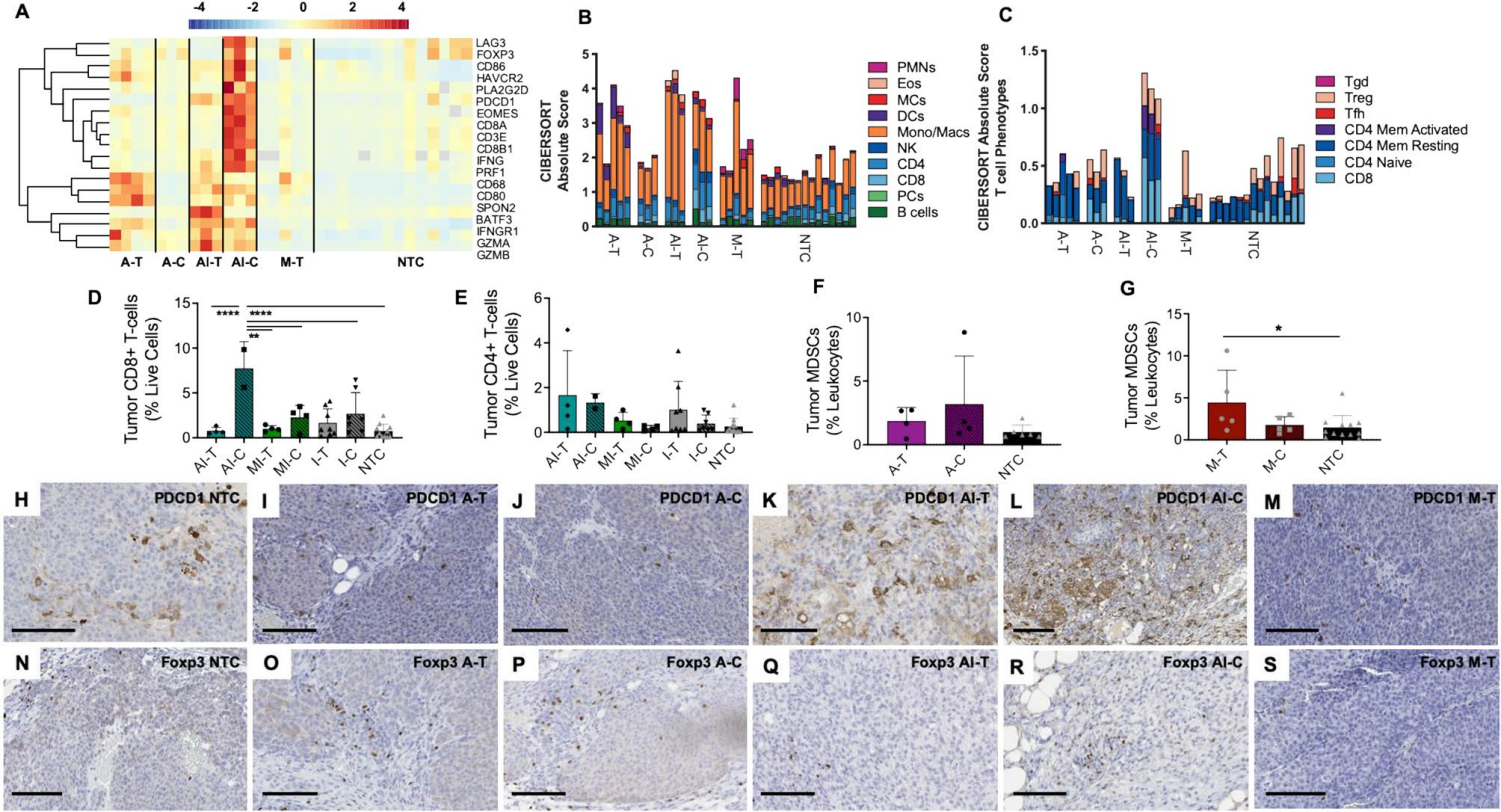
Thermal ablation stimulates a weak adaptive response that is augmented by TLR ligation and checkpoint modulation. (**A**) Z-scores of adaptive immune and T-cell response related genes for each treatment cohort. (**B**) CIBERSORTx-imputed absolute immune cell content. (**C**) CIBERSORTx-imputed T-cell phenotype distribution. Flow cytometry quantitation of (**D**) CD8^+^ and (**E**) CD4^+^ T-cells in tumors treated with a combined immunotherapy-HIFU protocol. Myeloid derived suppressor cells (MDSCs) as quantified with flow cytometry of the (**F**) thermal ablation and (**G**) mechanical ablation cohorts. Pdcd1 IHC stained sections of (**H**) an untreated control tumor, (**I**) a thermally-ablated tumor, (**J**) a distant tumor in an animal that received thermal ablation, (**K**) a tumor that received both thermal ablation and immunotherapy, (**L**) a distant tumor in an animal that received both thermal ablation and immunotherapy, and (**M**) a tumor treated with mechanical HIFU. FoxP3 IHC stained sections of (**N**) an untreated control tumor, (**O**) a thermally-ablated tumor, (**P**) a distant tumor in an animal that received thermal ablation, (**Q**) a tumor that received both thermal ablation and immunotherapy, (**R**) a distant tumor in an animal that received both thermal ablation and immunotherapy, and (**S**) a tumor treated with mechanical HIFU. Scale bar represents 100 µm in all cases. Time points for RNA-seq and IHC data are: 1-week post ablation for all cohorts that received thermal ablation, and 24 h post HIFU for cohorts that received mechanical HIFU. For flow cytometry studies, time points are 72 h for cohorts that received mechanical HIFU or immunotherapy in addition to thermal ablation and 1 week for cohorts that received thermal ablation alone. Data are expressed as mean ± SD. *p < 0.05, **p < 0.01, ****p < 0.0001 (ordinary one-way ANOVA with Tukey correction for multiple hypotheses). The heatmap was created in R Studio v1.2.5001 (https://rstudio.com).

In addition to macrophages, we hypothesized that tumor-infiltrating T cells increase as a result of ablative treatment. To test this hypothesis, we first investigated T cell activation markers within the transcriptome and found that T cell activation markers and adaptive immune genes were upregulated by thermal ablation in treated, but not distant tumors (Fig. 5A), which was expected of a localized treatment modality. The combined immunotherapy-ablation protocol further upregulated T cell activation markers such as *Gzma*, *Gzmb*, and *Batf3* in the directly-treated tumor compared to ablation alone. Additionally, the combined protocol extended the effect to distant sites, upregulating expression of adaptive immune genes such as *Eomes*, *Cd8a*, *Prf1*, and *Ifng* in distant tumors, extending the effect of localized ablation treatment to systemic effects. T cell and adaptive response pathways were likewise upregulated by the combination of ablation and immunotherapy (Supplementary Table S3) based on gene ontology enrichment analysis by DAVID. Mechanical HIFU did not alter adaptive immune gene expression compared to control. Based on the RNA-seq signature, macrophages were the predominant immune cell infiltrating tumors (Fig. 5B) but were underestimated by flow cytometry (Supplementary Fig. S6). Thermal ablation with immunotherapy increased CD8^+^ T cell infiltration into distant tumors (AI-C) (Fig. 5C).

CD8^+^ T cell infiltration at distant sites increased under the combined immunotherapy-HIFU protocols compared to the no treatment control cohort (Fig. 5D). However, only thermal ablation primed with immunotherapy improved CD8^+^ T cell recruitment compared to immunotherapy alone, indicating the possibility of synergistic effects from simultaneous DAMP and PAMP signaling. The thermal ablation-immunotherapy protocol also increased CD4^+^ T cell infiltration at both directly-treated and distant sites (Fig. 5E).  The impact of immunotherapy on the directly-treated tumor is further shown in Supplementary Fig. S7.

In addition to effector tumor infiltrating lymphocytes, immunosuppressive cell populations were induced by HIFU but in distinct patterns for thermal ablation compared to mechanical HIFU. Thermal ablation expanded immunosuppressive populations at distant sites while mechanical HIFU primarily enhanced these populations within directly-treated tumors. Tregs were enhanced in both directly-treated and distant tumors following thermally ablative therapies, while mechanical HIFU increased Tregs in directly-treated tumors (Fig. 5C). Both thermal ablation (Fig. 5F) and mechanical HIFU (Fig. 5G) increased the number of myeloid-derived suppressor cells (MDSCs) within the treated tumor compared to control (1.9-fold greater for thermal and 2.3-fold for mechanical), but the effect was only significant for mechanical ablation. Thermal ablation generated an ~ threefold increase in MDSC infiltration at distant tumor sites, while mechanical ablation did not enhance distant MDSC infiltration.

*Pdcd1* (PD-1) gene expression was decreased in HIFU monotherapy cohorts compared to control, but the combined immunotherapy-ablation protocol increased *Pdcd1* expression at both directly-treated and distant sites (Fig. 5A). On IHC, control tumors exhibited relatively scattered expression of Pdcd1 (Fig. 5H); thermal ablation reduced PD-1^+^ cells in both the directly-treated (Fig. 5I) and distant (Fig. 5J) tumors. Administration of CpG and anti-PD-1 followed by thermal ablation increased the number and infiltration of PD-1^+^ cells in both the directly-treated (Fig. 5K) and distant tumors (Fig. 5L). Pdcd1 expression was higher in AI-C as compared to AI-T tumors. Tumors treated with mechanical HIFU had similar PD-1 expression (Fig. 5M) as thermally-ablated tumors.

Foxp3 is primarily expressed on immunosuppressive CD4^+^ T cells, which suppress the activity of immune effector cells while simultaneously attenuating the inflammatory response. Treg infiltration has been associated with worse outcomes in some types of cancers (e.g. melanoma, breast, and ovarian) but paradoxically with improved tumor control in other cancers (e.g. head and neck and bladder cancers). Foxp3 expression, as observed on IHC, was induced by thermal ablation but reduced under the combined immunotherapy-ablation protocol. Control tumors had minimal Foxp3 expression (Fig. 5N) which was increased after thermal ablation in the directly-treated (Fig. 5O) and distant tumors (Fig. 5P). Priming with immunotherapy reduced the number of Foxp3^+^ cells in the directly-treated tumor (Fig. 5Q) compared to thermal ablation alone but marginally increased Foxp3^+^ cells in the distant tumor (Fig. 5R). Few Foxp3^+^ cells were observed following mechanical HIFU (Fig. 5S).

The combination of ablation and immunotherapy highly enriched multiple T cell activation, signaling and proliferation pathways (Supplementary Table S3). Thermal ablation enriched similar pathways to a lesser degree.

### Inflammatory cytokines and chemokines are upregulated by HIFU

**Figure 6:**
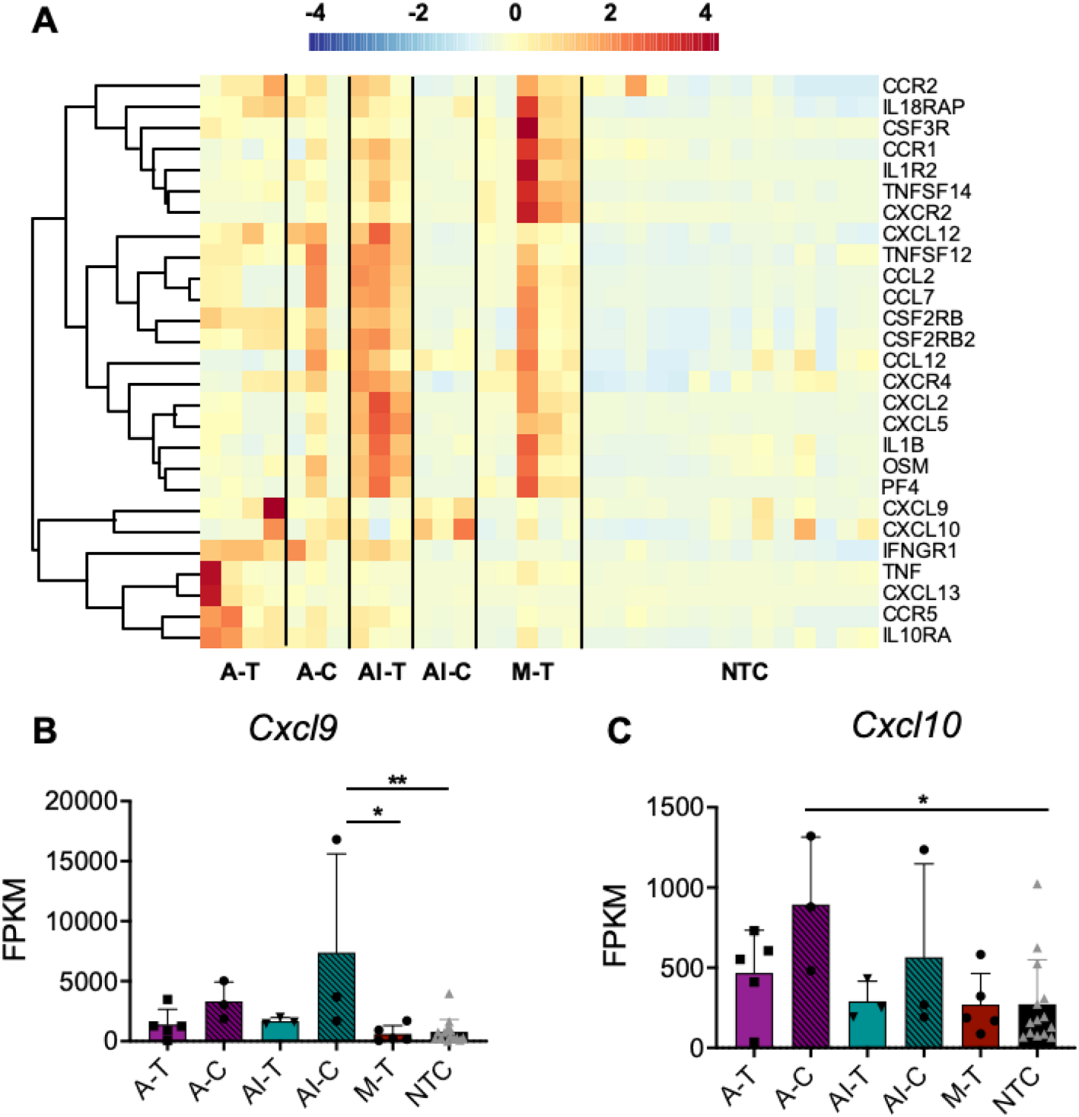
HIFU upregulates inflammatory cytokines and leukocyte signaling chemokines. (**A**) Heatmap visualization of Z-scores of leukocyte chemokines and cytokines for each treatment cohort. FPKM values for each cohort for (**B**) *Cxcl9* and (**C**) *Cxcl10*. Data are expressed as mean ± SD. *p < 0.05, **p < 0.01 (ordinary one-way ANOVA with Tukey correction for multiple hypotheses). Time points are: 1-week post ablation for all cohorts that received thermal ablation, and 24 h post HIFU for cohorts that received mechanical HIFU. The heatmap was created in R Studio v1.2.5001 (https://rstudio.com).

To further understand how the combination of HIFU and immunotherapy extended the overall immune stimulatory effect towards systemic lymphocyte activation and trafficking, we evaluated the genetic expression levels of inflammatory cytokines and chemokines. Across the combined immunotherapy-ablation treatment, mechanical HIFU and thermal ablation, T-cell chemotactic signaling chemokines and cytokines were enhanced in the treated tumors (Fig. 6A). The most enriched pathway across all treatment groups was neutrophil chemotaxis (GO:0030593) (Supplementary Table S4), which was reflected by strong upregulation of *Cxcl9*, the leukocyte chemotaxis signaling molecule, in the distant tumors of animals treated with the combined ablation-immunotherapy protocol (Fig. 6B). Chemokines related to leukocyte recruitment were upregulated in A-T tumors, which were largely distinct from those upregulated in M-T tumors. Thermal ablation upregulated *Cxcl10*, preferentially at distant tumor sites (Fig. 6C).

### Combining TLR9 ligation with ablation enhances the recruitment of B220^+^ cells

**Figure 7:**
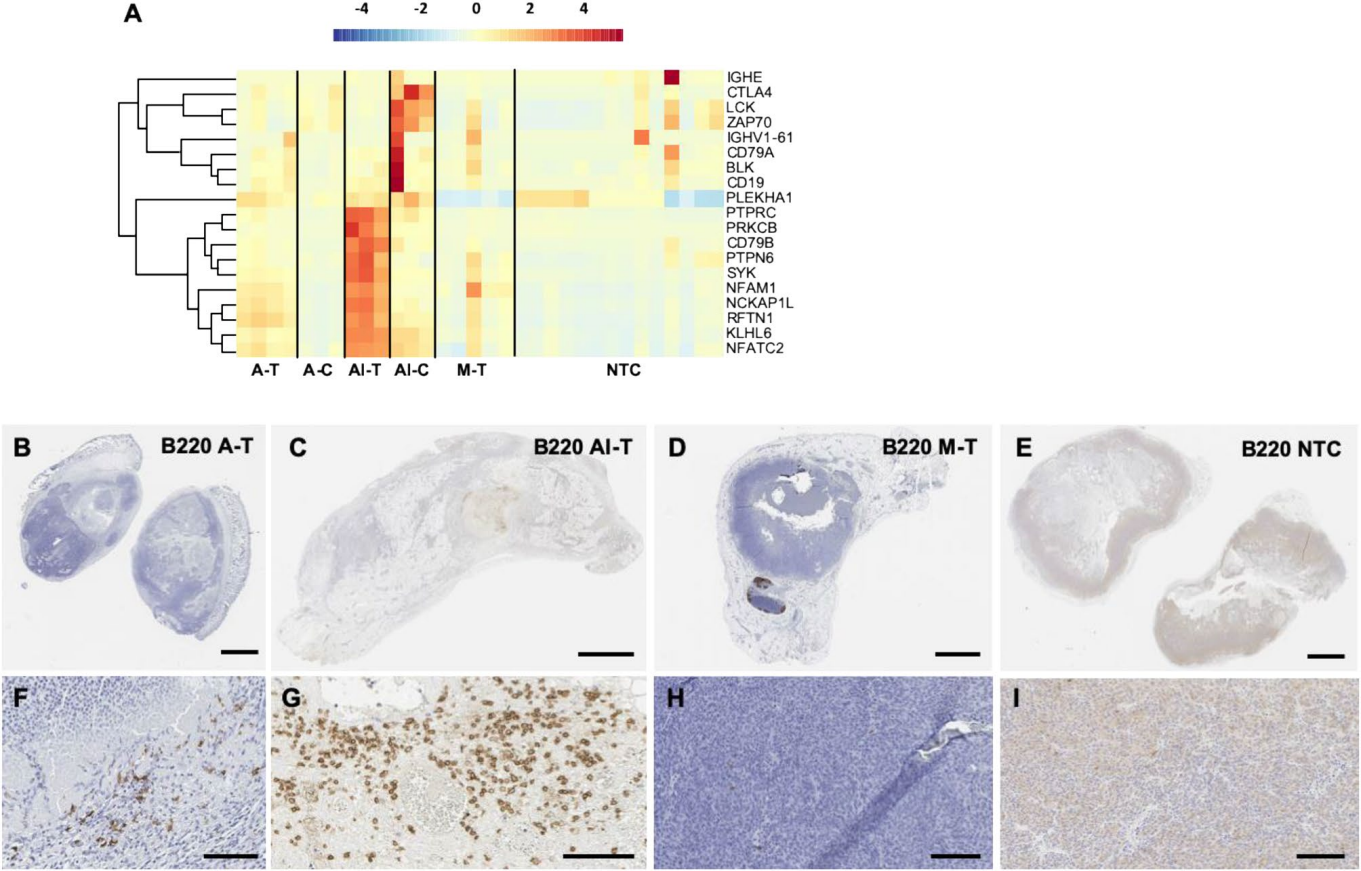
Thermal ablation recruits B220^+^ cells and upregulates pathways consistent with plasma-resident immune cell signaling and activation. (**A**) Z-scores of plasma immune cell-related genes for each treatment cohort. B220 stains of (**B**) thermally-ablated tumor, (**C**) tumor that received both thermal ablation and immunotherapy, (**D**) tumor treated with mechanical HIFU, and (**E**) untreated control tumor. Magnified views of the same B220-stained sections for (**F**) thermally-ablated tumor, (**G**) tumor that received both thermal ablation and immunotherapy, (**H**) tumor treated with mechanical HIFU, and (**I**) an untreated control tumor. Scale bar represents 2 mm in (**B–E**) and 100 µm in (**F**–**I**). Time points are: 1-week post ablation for all cohorts that received thermal ablation, and 24 h post HIFU for cohorts that received mechanical HIFU. The heatmap was created in R Studio v1.2.5001 (https://rstudio.com).

Since Il1β is related to the induction of humoral immunity^37^, we hypothesized that plasma-based immune cell gene expressions varied across treatment cohorts. To test this hypothesis, we first interrogated our mouse transcriptome for humoral-related gene expression and found that it was enhanced by thermal ablation as compared to control (Fig. 7A) in the directly-treated tumors. The addition of immunotherapy further increased genes associated with plasma-based immune cell receptor signaling in both the directly-treated and distant tumors (Supplementary Table S5). Thermal ablation upregulated *Siglech* in directly-treated tumors (Supplementary Fig. S8) compared to both control and AI-T tumors, while *Cd20* expression was significantly higher in AI-C tumors (Supplementary Fig. S8). IHC confirmed B220^+^ cell infiltration resulting from thermal ablation (Fig. 7B,F) and the further enhancement from priming with immunotherapy (Fig. 7C,G). Mechanical HIFU did not enhance B220^+^ cell infiltration (Fig. 7D,H) as compared with control tumors (Fig. 7E,I). On magnified sections, B220^+^ cells were localized to the rim of thermally-ablated tumors (Fig. 7F), but infiltrate within AI-T tumors (Fig. 7G). B220^+^ cells were not detected in tumors treated with mechanical HIFU (Fig. 7H) and control tumors (Fig. 7I) on magnified sections.

## Discussion

We have previously reported on the efficacy of a combined ablation-immunotherapy protocol in generating an adaptive immune response and reducing systemic tumor burden^15^. Here, we probe the basis of that response by examining innate immune receptors and cytokines that initiate the adaptive response and quantifying the immunomodulatory effects of thermal and mechanical HIFU alone. Thus, we can directly compare across two HIFU parameter sets and the effects of incorporating an immune agonist.

Thermal ablation has a broad effect on innate immune sensors and pathways. However, by itself, thermal ablation primarily releases DAMPs and produces a strong, sterile inflammatory response. Priming with an immune agonist attenuates *Il6* gene expression and enhances upregulation of innate receptors across multiple families, sensitizing the innate immune system to danger signals. When combined with innate immune agonists, tumor specific antigens released by ablation are processed by antigen presenting cells (APCs) such as macrophages and dendritic cells and cross-presented to T-cells, generating a systemic anti-tumor response. The strength and breadth of the innate immune response to HIFU offers multiple opportunities for synergy with agonists.

HIFU ablation of solid tumors is attractive due to its non-invasive nature and limited side-effect profile. Moreover, HIFU can be repeated as needed without regard to lifetime dose limits, unlike chemotherapy or radiation therapy. However, while HIFU can rapidly kill all viable tumor cells within a treated volume, as a monotherapy, HIFU is insufficient to generate a robust systemic anti-tumor response. In the mouse models we have studied, ablation alone does not result in any significant increase in survival^21^. Indeed, ablation releases DAMPs from damaged cells, recruiting neutrophils and monocytes to the ablated site, but in the absence of additional stimuli for pattern recognition receptors, DAMPs will be cleared and the leukocytes recruited by the initial inflammatory insult transition from acute inflammatory to reparative, or even generate a pro-tumor chronic inflammatory state via alteration in macrophage metabolism^38^. Nevertheless, both mechanical HIFU and thermal ablation can synergize with immunotherapy due to their immunomodulatory effects, both stimulatory and immunosuppressive at varying stages, and their ability to release substantial tumor-specific antigen. Ultimately, the efficacy of a combined ablation-immunotherapy protocol is highly dependent on the immunomodulatory agents and the timing of their administration relative to ablation.

We have previously reported on the importance of treatment sequence when incorporating ablation into an immunotherapy protocol^21^, where we found that a combined ablation-immunotherapy regimen was less effective than immunotherapy alone when the two treatments began simultaneously. However, the combined protocol significantly enhanced survival compared to immunotherapy alone when immunotherapy was initiated prior to ablation. In this study we found ablation broadly increases PRR expression across multiple sensor types. This would seem to suggest addition of innate agonists after ablation would result in increased survival. However, we hypothesize that the large increase in Il6 expression and the resulting sterile inflammatory response following ablation alters expression in PRR pathways. When pre-treated with immunotherapy, the increase in *Il6* expression is smaller following ablation (compared to ablation alone). We hypothesize this attenuation of *Il6* expression is one important component of generating an optimal anti-tumor immune response in the model studied here. Priming with immunotherapy substantially reduces the ablation-induced spike in *Il6* expression, perhaps via reducing expression of upstream *Il1β*. Furthermore, some DAMPs released by ablation, such as oxidized phosphorylcholine derivatives, can drive dendritic cells pre-exposed to TLR ligands into a hyperactive activation state, enhancing their antigen presentation capabilities^39,40^. Conversely, exposure to the same DAMPs prior to TLR ligation attenuated the inflammatory activity of DCs upon subsequent TLR ligation^41^, desensitized DCs to CD40-CD40L interaction and may limit migration of DCs to lymph nodes^42^.

Pre-treatment with innate immune agonists may slow the clearance of cellular debris generated by both thermal ablation or mechanical HIFU via generation of an inflammatory environment prior to and during the clearance process^43^. Thus, tumor specific antigen released by ablation may remain in situ and available for uptake by APCs longer when previously primed with immunotherapy. Aside from providing a reservoir of tumor antigen, ablation may also free amino and nucleic acids capable of inducing TLR-independent interferon production^44–47^, which may provide an additional mechanism for synergy. TLR tolerance can result in reduced type-1 IFN release after repeated ligation^48^, thus an alternative mechanism for type-1 IFN release, such as via HIFU, may be beneficial in those cases.

Thermal ablation increased MDSCs as a fraction of leukocytes within both the treated and distant tumors. Although Il1β can generate an adaptive anti-tumor immune response, following ablation it may produce a pro-tumor inflammatory response that ultimately results in MDSC recruitment and induction^49,50^. Furthermore, Il1β induces Il6 production, which promotes sustained, excessive inflammation, fostering a pro-tumor immune environment. Mechanical HIFU caused a similar increase in MDSCs in the directly-treated tumor, but mechanical HIFU did not increase MDSC expansion at distant sites. It has been suggested that release of tumor-derived microRNAs can induce MDSCs^51^, which may be one mechanism for the increase in MDSCs following destruction of tumor cells by mechanical HIFU. Furthermore, in the absence of immune adjuvants or co-stimulatory factors such as CD40 agonists, ablation alone may result in sub-optimal T cell priming and decreased T cell expansion^52,53^, and has been shown to induce resistance to checkpoint modulation^54^.

In addition to increased MDSC infiltration, thermal ablation increased the number and infiltration of FoxP3^+^ cells at both directly-treated and distant sites. Mechanical HIFU caused a slight decrease in FoxP3^+^ cells compared to control. Thermal and mechanical HIFU both reduced the number of PD-1^+^ cells on IHC compared to control, while pre-treatment with immunotherapy substantially increased PD-1^+^ cells in both directly-treated and distant tumors. The increase in PD-1 expression may relate to decreased *Il6* expression in cohorts that received immunotherapy given that Il6 blockade has been demonstrated to upregulate PD-L1^55^. We hypothesize that increased *Pdcd1* expression increases sensitivity to anti-PD-1 therapy, which was a component of the ablation-immunotherapy combined treatment protocol employed in this study.

Our study has multiple limitations. Based on flow cytometry, we chose to evaluate gene expression at 7 days following thermal ablation and 1 day after mechanical ablation. Our reasoning is that cell death from thermal ablation occurred over several days. Alternatively, the mechanical ablation resulted in immediate cell death with healing and tumor regrowth observed within a few days.

In our work, the same system (an MRgFUS system with a center frequency of 3 MHz) was used for all thermal and mechanical protocols. For thermal ablation, continuous wave insonation was applied at 3.1 MPa peak negative pressure (PNP), whereas for mechanical ablation a 16.9 MPa PNP was applied and the duty cycle was minimized such that any temperature increase was less than 2 °C. The resulting studies provide a starting point for the comparison of regimes; however, in the future, comparisons to the histotripsy protocols used in^56–59^ must also be made directly with the dedicated lower frequency equipment required for those studies. A disadvantage of such equipment (limiting a comparative study of thermal and mechanical mechanisms) is the larger insonified volume (relative to mouse tumors) and the incompatibility with MR guidance. The use of MRgFUS for the thermal ablation protocols is essential in order to quantify the temperature increase, assuring that a minimum temperature of 60 °C is achieved and the entire tumor is not exposed to this temperature for an extended period of time. Such an exposure can heat fix the tissue and reduce the immune effect. Such parameters will be the subject of future study. Finally, with bulk RNA-seq, we are unable to determine if enhanced/reduced transcript levels are the result of upregulation of expression of resident cells within the tumor or greater infiltration of cells constitutively expressing the examined genes at differing levels than tumor cells.

Overall, we found that both mechanical HIFU and thermal ablation rapidly kills viable tumor, with corresponding reductions in cancer and proliferation-related genes and pathways. Although HIFU does not appreciably activate an adaptive immune response, it induced inflammation via upregulation of *Il1β* and *Il6*, which leads to recruitment of MDSCs and subsequent tumor regrowth. Pre-treatment with an innate immune agonist, CpG in this case, and modulation of the PD-1/PD-L1 axis attenuates the excessive inflammatory response induced by ablation and overcomes rebound immune suppression.

## Methods

### Reagents

The TLR9 agonist, CpG 1826 (class B), was purchased from InvivoGen (San Diego, CA). Anti-mouse PD-1 (CD279) mAb (rat IgG2a, clone RMP1-14), was purchased from Bio X Cell (West Lebanon, NH).

### Ethics statement

All experiments and methods were performed in accordance with relevant guidelines and regulations. Specifically, all animal experiments were conducted with approval from the Stanford University Administrative Panel on Laboratory Animal Care (APLAC) and the University of California, Davis, (Davis, California) Institutional Animal Care and Use Committee (IACUC).

### Animal model

The *neu* exon deletion line (NDL), a syngeneic model of mammary adenocarcinoma, was obtained from the Alexander Borowsky Laboratory (UC Davis, Davis, CA). Four-week-old FVB/n female mice purchased from Charles River (Wilmington, MA) were transplanted with NDL tumor biopsies (~ 1 mm^3^) bilaterally into the fourth and ninth inguinal mammary fat pads. Approximately 21 days later, when tumors reached ~ 4 mm in longest dimension, mice were randomized into treatment groups.

### Therapeutic and sequencing protocols

A total of 167 mice were studied. For all studies, one tumor was directly-treated with either: immunotherapy alone (I-T), thermal ablation alone (A-T), mechanical ablation alone (M-T), thermal ablation primed with immunotherapy (AI-T), or mechanical ablation primed with immunotherapy (MI-T). RNA-seq data for the combination of thermal ablation and immunotherapy were reported previously in^15^ and are included for comparison. Distant (contralateral) tumors were also examined for each cohort (I-C, A-C, M-C, AI-C, and MI-C respectively). For flow cytometry quantification of immune cell populations, tumors were collected at 72 h and 7 days post thermal ablation (with and without immunotherapy), and at 24 h, 72 h, and 7 days post mechanical ablation. These studies provided the basis for the timing of the following sequencing studies. For RNA-seq studies, tumors were collected and sequenced 7 days following thermal ablation (with and without immunotherapy), or 24 h after mechanical HIFU. Our choice of the 24-h timepoint for mechanical HIFU was based on the ultrasound parameters we chose to employ and our experience with their biological effects and immunohistochemistry studies that we performed at 2 h, 24 h, 48 h, 72 h, and 7 days post mechanical ablation (Supplementary Fig. S1 showing tissue morphology on H&E stained sections); namely, cell death/destruction was significantly more rapid with mechanical ablation than with thermal ablation. Following thermal ablation, cells slightly distanced from the focal zone receive a lethal thermal dose due to heat diffusion but may take tens of hours to die. Consequently, cell debris is more rapidly released after mechanical ablation than after thermal ablation.

Animals receiving ablation and immunotherapy were primed with immunotherapy prior to ablation as follows: 100 μg CpG injected intratumorally (i.t.) on days 21, 24, 28, and 30; 200 μg anti-PD-1 injected intraperitoneally (i.p.) on days 21, 28, and 34, and ablation was performed on day 30.

### MRgFUS ablation protocol

All ablations were performed under MR guidance on a Bruker BioSpec 7T small animal MR system (Bruker Biospin) with core body temperature monitoring using a 16-element annular array transducer operating at 3 MHz (Imasonic SAS)^60^. Acoustic pressure was calibrated with a fiber optic hydrophone (HFO690, Onda Corp.) in a degassed water tank under free-field conditions. Prior to ablation, mice were given 0.05–0.1 mg/kg buprenorphine subcutaneously (s.c.) and 0.05 mmol/kg gadoteridol (Bracco Imaging) i.p. and imaged with a T1w RARE (TE/TR = 12.5/750 ms, FOV = 4.8 cm × 4.8 cm, MTX = 256 × 256, ST/SI = 1/1 mm, 17 slices) sequence for tumor localization and treatment planning. Tumors were then ablated and temperature was monitored in real time via the MR proton resonance frequency shift using Thermoguide Software (Image Guided Therapy), with α = − 0.0101 ppm/°C, TE/TR = 4.5/21 ms^5^.

For thermal ablation, continuous wave (CW) insonation was employed at 3.1 MPa in a circular pattern (diameter of 2 mm, scan speed of 1 revolution per second) until the targeted volume reached at least 60 °C and a thermal dose in cumulative equivalent minutes at 43 degrees (CEM43) of more than 5000 was achieved^15^.

For mechanical ablation, tumors were ablated in a grid pattern with individual points separated by 0.5 mm, treating the entirety of the tumor with the exception of a ~ 0.5–0.75 mm rim. Each point of the grid pattern was insonated for 5 ms at 16.9 MPa (peak negative pressure), 95.3 MPa (peak positive pressure), with a duty cycle of 0.5%. The duty cycle was chosen to minimize thermal effects and maintain temperature elevations ≤ 2 °C. The grid pattern was repeated 10 times.

### Histology and immunohistochemistry

Tissues for microscopic analysis were prepared as previously described^15^. Briefly, tissues were fixed in 10% buffered formalin for 24 h or longer, followed with 70% ethanol overnight before further processing. A Tissue-Tek VIP autoprocessor (Sakura, Torrance, CA) was used to process samples for paraffin-embedding. Tissue blocks were then sectioned to 4 μm, and the sections were mounted on glass slides. Hematoxylin and Eosin (H&E) staining was performed at the University of California, Davis, Department of Pathology and Laboratory Medicine. For staining of cytotoxic T cells, the rat anti-mouse CD8a primary antibody was used (1:500; 14-0808, eBiosciences). All IHC was performed manually without the use of an automated immunostainer and using the ABC method. Antigen retrieval was performed using a Decloaking Chamber (Biocare Medical, Concord, CA) with citrate buffer at pH 6.0, 125 °C and pressurized to 15 psi. The total time slides were in the chamber was 45 min. Incubation with the primary antibody was performed at ambient temperature overnight in a humidified chamber. Normal goat serum was used for blocking. Biotinylated goat anti-rat (1:500; Vector Labs, Burlingame, CA) was the secondary antibody used with a Vectastain ABC Kit Elite and a Peroxidase Substrate Kit DAB (both from Vector Labs) used for the amplification and visualization of signal, respectively. Mouse spleen was used as a positive control. Stained slides were scanned on an AT2 Scanscope (Leica Biosystems) and digital images viewed using the ImageScope program (Leica Biosystems).

### Flow cytometry antibodies

Flow cytometry was performed with mouse-specific fluorochrome-conjugated monoclonal antibodies (mAbs) as previously described^15^. Briefly, Pacific blue (PB)-anti-CD45 (30-F11), fluorescein isothiocyanate (FITC)-anti-F4/80 (BM8), phycoerythrin (PE)-Cy7-anti-CD11c (N418), PE-Cy7-anti-CD3 (145-2C11), allophycocyanin (APC)-Cy7-anti-CD11b (M1/70), Alexa Fluor (AF)-700-anti-Ly6G/Ly6C (Gr-1, RB6-8C5), and AF700-anti-CD8a (53–6.7) were purchased from BioLegend (San Diego, CA). FITC-anti-CD4 (GK1.5) and APC-anti-CD206 (C068C2) were purchased from BD Biosciences (San Jose, CA). PE-Cy5-anti-MHCII (M5/114.15.2) and PE-anti-CD86 (GL1) were purchased from eBioscience (San Diego, CA). Negative fluorescence-minus-one (FMO) control staining was performed with isotype-matched mouse, rat or hamster IgG mAbs, and nonspecific binding was blocked with the FcγIII/II receptor-mediated anti-CD16/CD32 antibody (2.4G2) from BD Biosciences.

### Flow cytometry

Mice were sacrificed at indicated time points following treatment, and tissues (tumors, tumor draining lymph nodes, spleen, and blood) were collected and processed by mechanical and enzymatic disruption to single-cell suspensions for immune cell profiling via flow cytometry. Briefly, after euthanizing tumor-burdened mice, bilateral tumors were dissected and stored in 1 mL of DMEM on ice. For mechanical disruption, tumors were then finely minced using dissection scissors. Following this, the tumor solution was enzymatically digested by adding 40 μL of Liberase DL (28 U/mL stock), 80 μL of Liberase TL (14 U/mL stock), and 40 μL of DNase I (15 mg/mL stock) and incubated at 37 °C under continuous rotation^61^. After incubation, the digested tumor solution was triturated, passed through a 70 μm cell strainer, and washed with several mL of DMEM + 10% FBS. The solution was spun at 300G for 5 min at 4 °C and resuspended in 1 mL of fresh DMEM + 10% FBS for subsequent cell counting and flow cytometry antibody staining.

Live-dead cell staining with the LIVE/DEAD Fixable Aqua Dead Cell Stain Kit (Invitrogen, Carlsbad, CA) was performed according to the manufacturer’s instructions prior to all other antibody staining in order to exclude dead cells from analysis. Antibody panel combinations used to distinguish immune cell populations were CD45^+^ (leukocytes) plus the following: CD11b^+^, F4/80^+^, Gr-1^−^ (macrophages); CD11b^+^, F4/80^+^, Gr-1^−^, CD86^+^, CD206^−^ (M1 macrophages); CD11b^+^, F4/80^+^, Gr-1^−^, CD86^−^, CD206^+^ (M2 macrophages); CD11b^+^, F4/80^+^, Gr-1^−^, CD86^+^, CD206^+^ (M1/M2 macrophages); CD11b^+^, F4/80^+^, Gr-1^−^, CD86^−^, CD206^−^ (non-M1/M2 macrophages); CD11c^+^, MHCII^+^, F4/80^−^ (dendritic cells); CD11b^+^, Ly6G/Ly6C^+^ (myeloid derived suppressor cells); CD3^+^ (T-cells); CD3^+^, CD4^+^ (CD4^+^ T-cells); CD3^+^, CD8^+^ (CD8^+^ T-cells). Cell samples were fixed in Cytofix buffer (BD Biosciences), diluted to 1% paraformaldehyde (PFA) in PBS−/−, and run within 24 h on an LSRII flow cytometer (BD Biosciences). All data were analyzed using FlowJo v10 software (TreeStar).

### RNA isolation

Snap-frozen NDL tumors were submitted to the UC Davis Comprehensive Cancer Center’s Genomics Shared Resource for isolation of total cellular RNA and subsequent RNA-seq analysis. Total cellular RNA was isolated using the TRIzol Reagent (Invitrogen) using a modified protocol that incorporates an additional extraction with phenol/chloroform/isoamyl alcohol (25:24:1, pH 4.3) followed by clean-up with an RNeasy spin column (Qiagen). RNA concentration and purity were assessed with a NanoDrop 1000 Spectrophotometer (Thermo Scientific) and quality assessments were made using an Agilent 2100 Bioanalyzer (Agilent Technologies, Santa Clara, CA).

### Directional RNA-seq library preparation and next-generation sequencing

Indexed RNA-seq libraries were prepared using the NEBNext Ultra Directional RNA Library Prep Kit (New England BioLabs, Ipswich, MA), according to the manufacturer’s standard protocol as previously described^15^. Briefly, poly(A) mRNA was captured from total RNA (100 ng) by binding to magnetic oligo(dT)_25_ beads, fragmented, and then double-stranded cDNA generated by random-primed first-strand synthesis and second strand synthesis in the presence of dUTP for strand marking^62,63^. The double-stranded cDNA was then end repaired and 3′-dA tailed, followed by ligation of an Illumina-compatible adaptor and subsequent uridine excision with the USER (Uracil-Specific Excision Reagent) enzyme. The libraries were then indexed and enriched by high-fidelity PCR amplification (15 cycles) with i7 primer/index primers and Q5 High-Fidelity DNA Polymerase. Subsequently, libraries were pooled for multiplex sequencing on an Illumina HiSeq 4000 System (150-bp, paired-end, ~ 25–30 × 10^6^ reads per sample).

### NGS data processing and analysis

RNA-seq data was analyzed using a STAR-StringTie-Cufflinks pipeline. Raw sequence reads (FASTQ format) were mapped to the reference mouse genome assembly (GENCODE, GRCm38, release 05/2017) using STAR (Spliced Transcripts Alignment to a Reference) software with a 2-pass alignment approach with an initial alignment to detect novel junctions and to insert these junctions into the genome index, followed by a second pass to re-align reads using both annotated (GENCODE, Release M14, GRCm38.p5) and novel junctions^64^. Mapped reads were then passed onto StringTie for transcript assembly^65^. Subsequently, transcript-level expression was quantified with Cufflinks tools (e.g. Cuffquant, Cuffnorm) to yield normalized expression as FPKM (fragments per kilobase of transcript per million fragments mapped reads) and test for differential expression (Cuffdiff)^66^.

Cuffnorm files were imported to MATLAB for processing and analysis. Z-scores were calculated using raw FPKM values across all presented samples for a single gene in R. Principal component analysis was performed in R. Gene set enrichment analysis was performed on the set of all differentially expressed genes for each Cuffdiff analysis (i.e. genes with a differential expression at p < 0.01) using DAVID’s Functional Annotation Tool. Specifically, the GO Biological Processes and the KEGG Pathways were investigated^67^.

### CIBERSORTx

Bulk RNA-seq data was input to CIBERSORTx^68^ at https://cibersortx.stanford.edu, which imputed immune cell fractions.

### Statistical analysis

Statistical analyses were performed using Prism 8 software (GraphPad Software Inc). Results are presented as mean ± SD, unless otherwise indicated. One-way ANOVA was performed for all analyses of three or more groups followed by a with Tukey correction for multiple hypotheses in GraphPad Prism. Analysis of differences between two groups was performed using an unpaired t-test assuming unequal variance. p values less than 0.05 were considered significant.

## Supplementary Information

Supplementary Information.

## References

[1] Ning Z (2019). HIFU is safe, effective, and feasible in pancreatic cancer patients: A monocentric retrospective study among 523 patients. Onco Targets Ther..

[2] Strunk HM (2016). Clinical use of high-intensity focused ultrasound (HIFU) for tumor and pain reduction in advanced pancreatic cancer. Rofo.

[3] Khokhlova TD, Hwang JH (2016). HIFU for palliative treatment of pancreatic cancer. Adv Exp. Med. Biol..

[4] de Senneville BD, Moonen C, Ries M (2016). MRI-guided HIFU methods for the ablation of liver and renal cancers. Adv. Exp. Med. Biol..

[5] Fite BZ (2012). Magnetic resonance thermometry at 7T for real-time monitoring and correction of ultrasound induced mild hyperthermia. PLoS ONE.

[6] Kohler MO (2009). Volumetric HIFU ablation under 3D guidance of rapid MRI thermometry. Med. Phys..

[7] Gaur P, Grissom WA (2015). Accelerated MRI thermometry by direct estimation of temperature from undersampled k-space data. Magn. Reson. Med..

[8] Rieke V, Butts Pauly K (2008). MR thermometry. J. Magn. Reson. Imaging.

[9] Maxwell AD (2011). Cavitation clouds created by shock scattering from bubbles during histotripsy. J. Acoust. Soc. Am..

[10] Simon JC (2011). Miniature acoustic fountain mechanism for tissue emulsification during millisecond boiling in high intensity focused ultrasound fields. J. Acoust. Soc. Am..

[11] Hall TL (2007). Histotripsy of rabbit renal tissue in vivo: Temporal histologic trends. J. Endourol..

[12] Hoogenboom M (2016). In vivo MR guided boiling histotripsy in a mouse tumor model evaluated by MRI and histopathology. NMR Biomed..

[13] Lu P (2009). Increased infiltration of activated tumor-infiltrating lymphocytes after high intensity focused ultrasound ablation of human breast cancer. Surgery.

[14] Hu Z (2005). Release of endogenous danger signals from HIFU-treated tumor cells and their stimulatory effects on APCs. Biochem. Biophys. Res. Commun..

[15] Chavez M (2018). Distinct immune signatures in directly treated and distant tumors result from TLR adjuvants and focal ablation. Theranostics.

[16] van den Bijgaart RJ (2017). Thermal and mechanical high-intensity focused ultrasound: Perspectives on tumor ablation, immune effects and combination strategies. Cancer Immunol. Immunother..

[17] Reits EA (2006). Radiation modulates the peptide repertoire, enhances MHC class I expression, and induces successful antitumor immunotherapy. J. Exp. Med..

[18] Takahashi Y (2016). Optimized magnitude of cryosurgery facilitating anti-tumor immunoreaction in a mouse model of Lewis lung cancer. Cancer Immunol. Immunother..

[19] Kroemer G, Galluzzi L, Kepp O, Zitvogel L (2013). Immunogenic cell death in cancer therapy. Annu. Rev. Immunol..

[20] Wu F (2004). Activated anti-tumor immunity in cancer patients after high intensity focused ultrasound ablation. Ultrasound Med. Biol..

[21] Silvestrini MT (2017). Priming is key to effective incorporation of image-guided thermal ablation into immunotherapy protocols. JCI Insight.

[22] Iwasaki A, Medzhitov R (2010). Regulation of adaptive immunity by the innate immune system. Science.

[23] Nathan C (2002). Points of control in inflammation. Nature.

[24] Messenheimer DJ (2017). Timing of PD-1 blockade is critical to effective combination immunotherapy with anti-OX40. Clin. Cancer Res..

[25] Hotz C, Bourquin C (2012). Systemic cancer immunotherapy with Toll-like receptor 7 agonists: Timing is everything. Oncoimmunology.

[26] Sato-Kaneko F (2017). Combination immunotherapy with TLR agonists and checkpoint inhibitors suppresses head and neck cancer. JCI Insight..

[27] Lundt JE (2017). Non-invasive, rapid ablation of tissue volume using histotripsy. Ultrasound Med. Biol..

[28] Hurme T, Kalimo H, Sandberg M, Lehto M, Vuorio E (1991). Localization of type I and III collagen and fibronectin production in injured gastrocnemius muscle. Lab. Investig..

[29] Wang Y (2016). Collagen type III alpha1 as a useful diagnostic immunohistochemical marker for fibroepithelial lesions of the breast. Hum. Pathol..

[30] Kanneganti TD (2010). Central roles of NLRs and inflammasomes in viral infection. Nat. Rev. Immunol..

[31] Barton GM, Medzhitov R (2002). Toll-like receptors and their ligands. Curr. Top. Microbiol. Immunol..

[32] He Y, Hara H, Nunez G (2016). Mechanism and regulation of NLRP3 inflammasome activation. Trends Biochem. Sci..

[33] Dinarello CA (2018). Overview of the IL-1 family in innate inflammation and acquired immunity. Immunol. Rev..

[34] Kanehisa M (2019). Toward understanding the origin and evolution of cellular organisms. Protein Sci..

[35] Kanehisa M, Goto S (2000). KEGG: Kyoto encyclopedia of genes and genomes. Nucleic Acids Res..

[36] Liu M (2019). Metabolic rewiring of macrophages by CpG potentiates clearance of cancer cells and overcomes tumor-expressed CD47-mediated 'don't-eat-me' signal. Nat. Immunol..

[37] Nakae S, Asano M, Horai R, Iwakura Y (2001). Interleukin-1 beta, but not interleukin-1 alpha, is required for T-cell-dependent antibody production. Immunology.

[38] Di Gioia M (2020). Endogenous oxidized phospholipids reprogram cellular metabolism and boost hyperinflammation. Nat. Immunol..

[39] Zanoni I (2016). An endogenous caspase-11 ligand elicits interleukin-1 release from living dendritic cells. Science.

[40] Zanoni I, Tan Y, Di Gioia M, Springstead JR, Kagan JC (2017). By capturing Inflammatory lipids released from dying cells, the receptor CD14 induces inflammasome-dependent phagocyte hyperactivation. Immunity.

[41] Bochkov VN (2002). Protective role of phospholipid oxidation products in endotoxin-induced tissue damage. Nature.

[42] Bluml S (2005). Oxidized phospholipids negatively regulate dendritic cell maturation induced by TLRs and CD40. J. Immunol..

[43] Munoz LE, Lauber K, Schiller M, Manfredi AA, Herrmann M (2010). The role of defective clearance of apoptotic cells in systemic autoimmunity. Nat. Rev. Rheumatol..

[44] Baccala R, Hoebe K, Kono DH, Beutler B, Theofilopoulos AN (2007). TLR-dependent and TLR-independent pathways of type I interferon induction in systemic autoimmunity. Nat. Med..

[45] Andrade F, Casciola-Rosen L, Rosen A (2000). Apoptosis in systemic lupus erythematosus. Clinical implications. Rheum. Dis. Clin. N. Am..

[46] Janssen E (2006). Efficient T cell activation via a Toll-Interleukin 1 receptor-independent pathway. Immunity.

[47] Fritz JH, Ferrero RL, Philpott DJ, Girardin SE (2006). Nod-like proteins in immunity, inflammation and disease. Nat. Immunol..

[48] Koga-Yamakawa E (2015). TLR7 tolerance is independent of the type I IFN pathway and leads to loss of anti-tumor efficacy in mice. Cancer Immunol. Immunother..

[49] Kaplanov I (2019). Blocking IL-1beta reverses the immunosuppression in mouse breast cancer and synergizes with anti-PD-1 for tumor abrogation. Proc. Natl. Acad. Sci. U.S.A..

[50] Voronov E, Apte RN (2017). Targeting the tumor microenvironment by intervention in interleukin-1 biology. Curr. Pharm. Des..

[51] Huber V (2018). Tumor-derived microRNAs induce myeloid suppressor cells and predict immunotherapy resistance in melanoma. J. Clin. Investig..

[52] Sanchez PJ, McWilliams JA, Haluszczak C, Yagita H, Kedl RM (2007). Combined TLR/CD40 stimulation mediates potent cellular immunity by regulating dendritic cell expression of CD70 in vivo. J. Immunol..

[53] Byrne KT, Vonderheide RH (2016). CD40 stimulation obviates innate sensors and drives T cell immunity in cancer. Cell Rep..

[54] Verma V (2019). PD-1 blockade in subprimed CD8 cells induces dysfunctional PD-1(+)CD38(hi) cells and anti-PD-1 resistance. Nat. Immunol..

[55] Tsukamoto H (2018). Combined blockade of IL6 and PD-1/PD-L1 signaling abrogates mutual regulation of their immunosuppressive effects in the tumor microenvironment. Cancer Res..

[56] Pahk KJ (2019). Boiling histotripsy-induced partial mechanical ablation modulates tumour microenvironment by promoting immunogenic cell death of cancers. Sci. Rep..

[57] Hendricks AD (2019). Histotripsy initiates local and systemic immunological response and reduces tumor burden in breast cancer. J. Immunol..

[58] Qu S (2020). Non-thermal histotripsy tumor ablation promotes abscopal immune responses that enhance cancer immunotherapy. J. Immunother. Cancer.

[59] Schade GR (2019). Boiling histotripsy ablation of renal cell carcinoma in the eker rat promotes a systemic inflammatory response. Ultrasound Med. Biol..

[60] Wong AW (2016). Ultrasound ablation enhances drug accumulation and survival in mammary carcinoma models. J. Clin. Investig..

[61] Cassetta L (2016). Isolation of mouse and human tumor-associated macrophages. Adv. Exp Med Biol.

[62] Borodina T, Adjaye J, Sultan M (2011). A strand-specific library preparation protocol for RNA sequencing. Methods Enzymol..

[63] Levin JZ (2010). Comprehensive comparative analysis of strand-specific RNA sequencing methods. Nat Methods.

[64] Dobin A, Gingeras TR (2015). Mapping RNA-seq reads with STAR. Curr. Protoc. Bioinform..

[65] Pertea M (2015). StringTie enables improved reconstruction of a transcriptome from RNA-seq reads. Nat. Biotechnol..

[66] Trapnell C (2012). Differential gene and transcript expression analysis of RNA-seq experiments with TopHat and Cufflinks. Nat. Protoc..

[67] da Huang W, Sherman BT, Lempicki RA (2009). Systematic and integrative analysis of large gene lists using DAVID bioinformatics resources. Nat. Protoc..

[68] Newman AM (2019). Determining cell type abundance and expression from bulk tissues with digital cytometry. Nat. Biotechnol..

