# Supplementary material for: Immune modulation resulting from MR-guided high intensity focused ultrasound in a model of murine breast cancer
Source: Sci Rep. 2021 Jan 13;11:927. doi: 10.1038/s41598-020-80135-1 (PMC7806949; doi:10.1038/s41598-020-80135-1)
Supplement: Supplementary file 1 — Supplementary Information. [file 41598_2020_80135_MOESM1_ESM.pdf]

Supplementary Figures and Tables

for

Immune modulation resulting from MR-guided high intensity focused ultrasound in a model of murine breast cancer

Brett Z. Fite, James Wang, Aris Kare, Asaf Ilovitsh, Michael Chavez, Tali Ilovitsh, Nisi Zhang, Weiyu Chen, Elise Robinson, Hua Zhang, Azadeh Kheirolomoom, Matthew T. Silvestrini, Elizabeth S. Ingham, Lisa M. Mahakian, Sarah M. Tam, Ryan R. Davis, Clifford G. Tepper, Alexander D. Borowsky, Katherine W. Ferrara

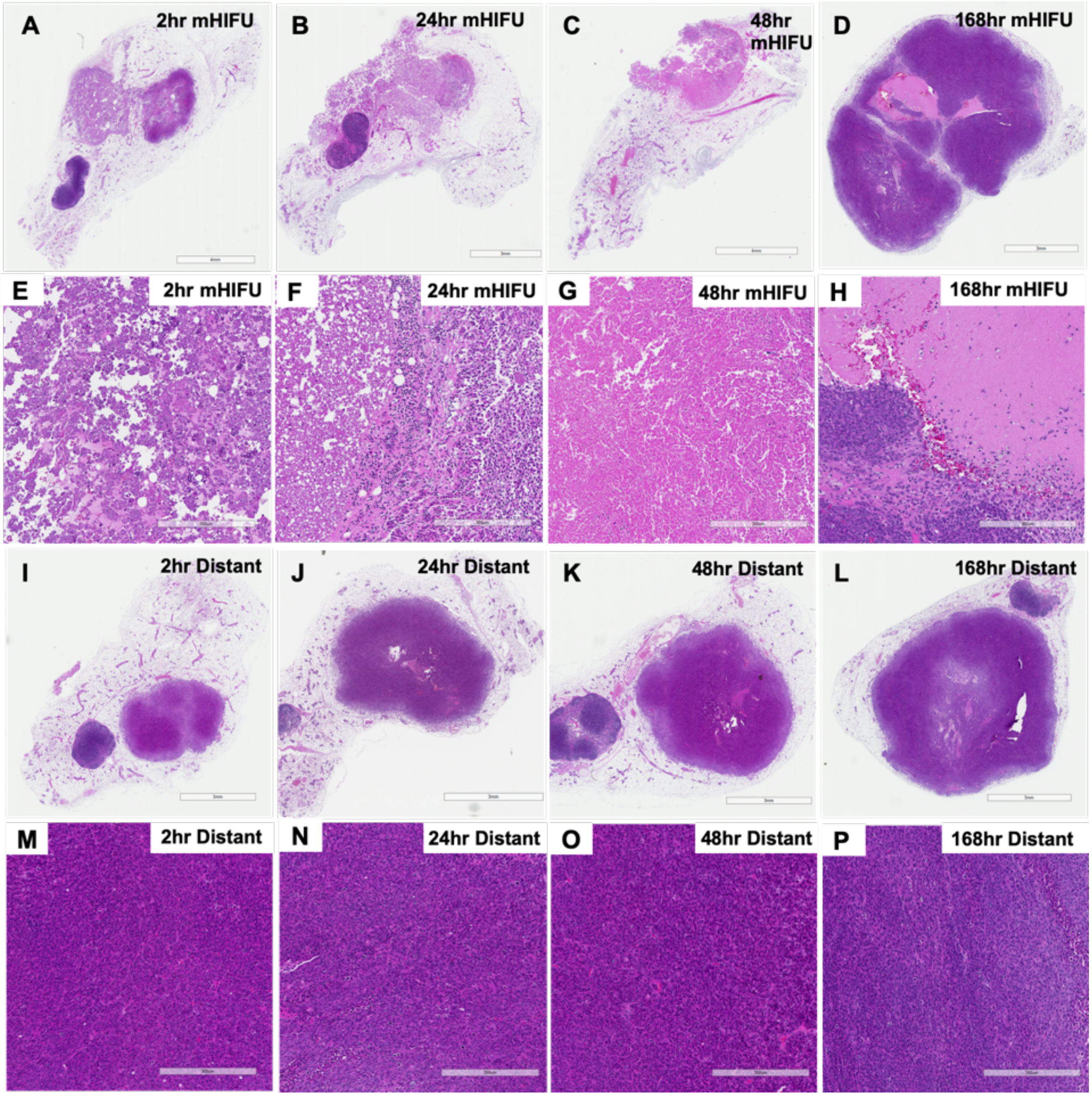

Supplementary Figure S1. Mechanical HIFU causes tissue discohesion. H&E stained directly-treated tumor sections at (A) 2 h, (B) 24 h, (C) 48 h, and (D) one week post mechanical ablation and (E-H) magnified views of the same sections as in the top row. H&E stained sections of the corresponding distant tumors at (I) 2 h, (J) 24 h, (K) 48 h, and (L) one week along with (M-P) magnified views of the same distant tumors.

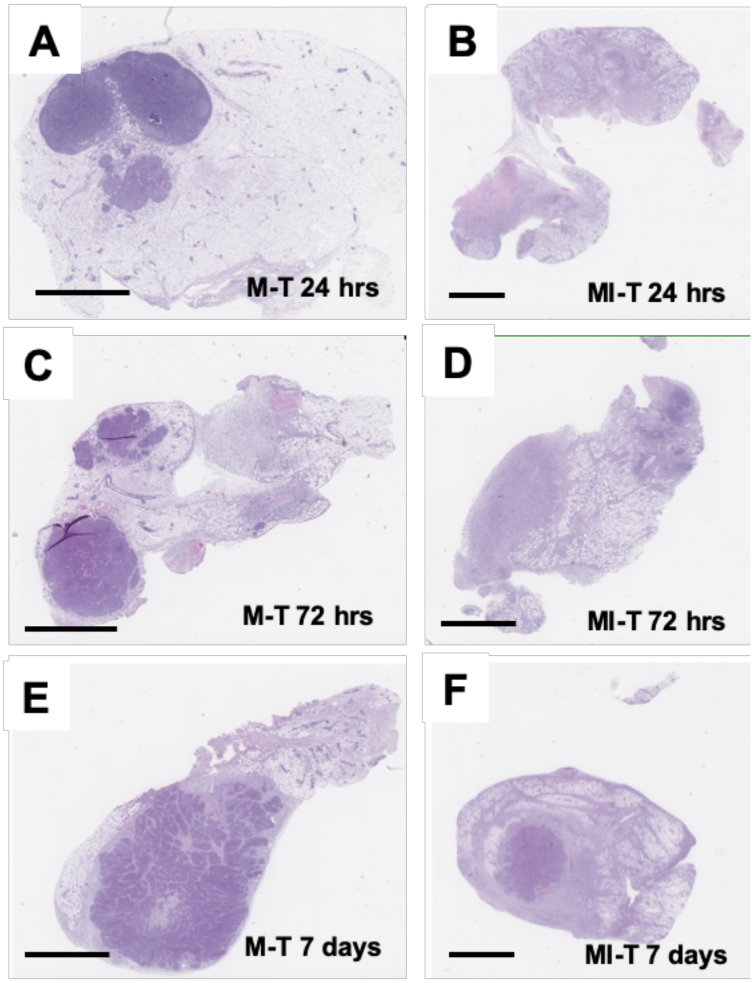

Supplementary Figure S2. Mechanical HIFU causes cell death, pretreatment with immunotherapy enhances this effect. At 24hr post mechanical HIFU, (A) tumors remain viable on H&E for tumors treated with a monotherapy. Addition of immunotherapy (B) results in extensive loss of tumor cell viability. At 72hr post mechanical HIFU, there is reduced viability in (C) mHIFU only that is further reduced in (D) the combination of mHIFU and immunotherapy. At 7 days post mechanical HIFU (E) mHIFU only tumors have increased in size while (F) treatments in the combination cohort have little viable tumor remaining. All scale bars represent 2mm.

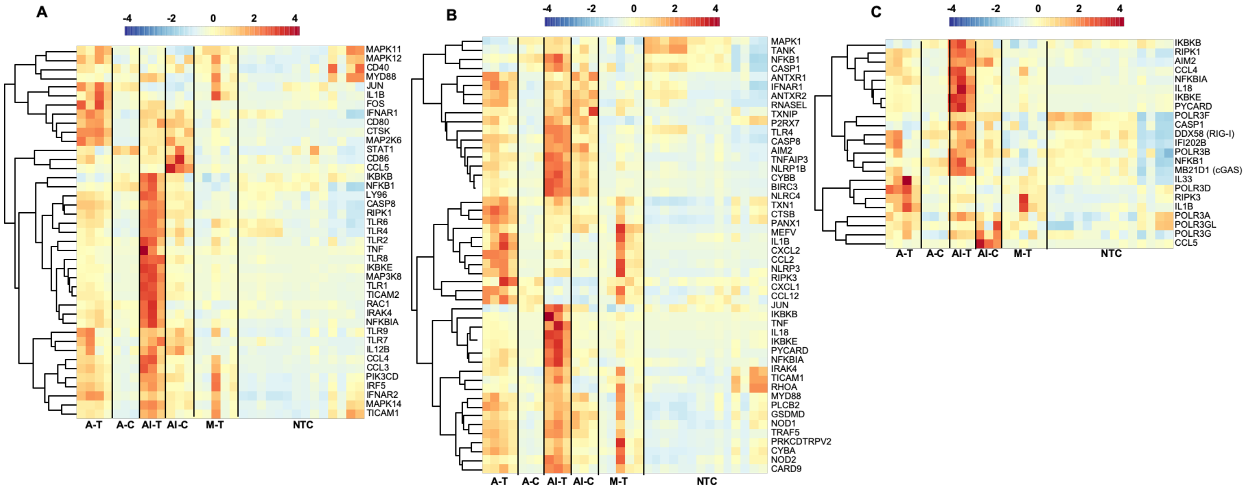

Supplementary Figure S3. Innate immune signaling pathways. Heatmap visualization of Z-scores for (A) Toll-like receptor signaling pathway, (B) NOD-like receptor signaling pathway, and (C) cytosolic DNA sensing and signaling pathway. The heatmaps were created in R Studio v1.2.5001 (https://rstudio.com).

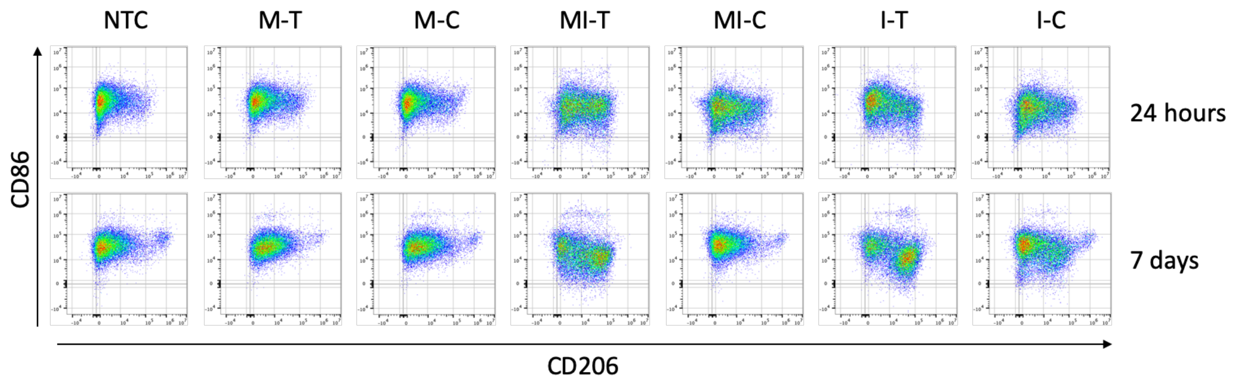

Supplementary Figure S4. Mechanical HIFU combined with immunotherapy alters populations of CD86^{+} CD206^{+} macrophages. Cd206^{low} macrophage populations are reduced by the combined mHIFU-immunotherapy treatment in the treated tumor at both 24hr and 7 days post HIFU. Each plot shows an equal representation of 20k cells for each cohort. The plots were created in FlowJo v10.7 (https://www.flowjo.com).

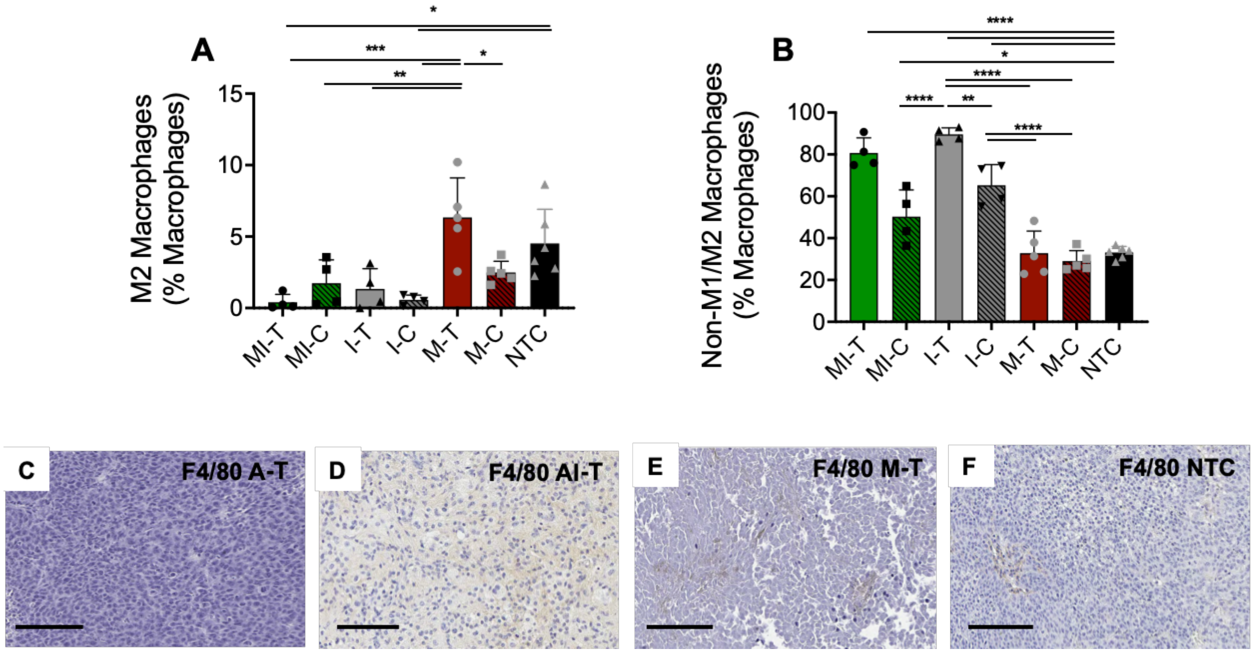

Supplementary Figure S5. Immunotherapy alters macrophage polarization of tumors treated with mechanical HIFU. Flow cytometry quantification of (A) M2 polarized macrophages and (B) macrophages with neither M1 nor M2 polarization as a percentage of total macrophages within the tumor. F4/80 IHC stained sections of (C) a thermally-ablated tumor, (D) a tumor that received both thermal ablation and immunotherapy, (E) a tumor treated with mechanical HIFU, and (F) an untreated control tumor. Scale bar represents 100 μm in all cases. Time points are: one-week post ablation for all cohorts that received thermal ablation, and 24 hours post HIFU for cohorts that received mechanical HIFU. Data are expressed as mean ± SD. *$p<0.05$, **$p<0.01$, ***$p<0.001$, ****$p<0.0001$ (ordinary one-way ANOVA with Tukey correction for multiple hypotheses).

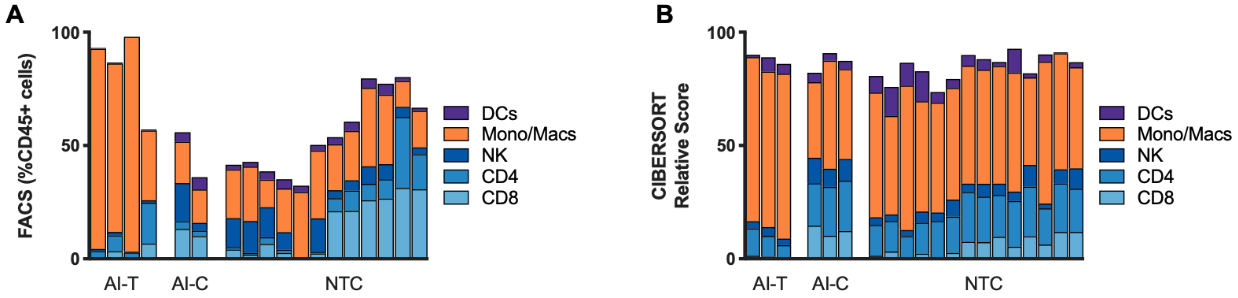

Supplementary Figure S6. Ablation increases macrophage infiltration. Myeloid cells were underestimated in (A) flow cytometry quantification compared to (B) CIBERSORT imputed proportions. However, both methodologies validated increases in tumor macrophages following ablation.

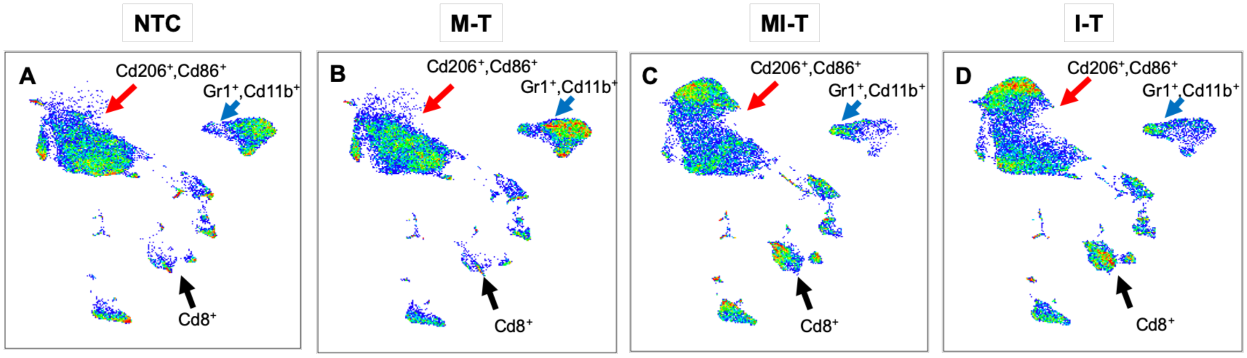

Supplementary Figure S7. The combination of mechanical ablation and immunotherapy enrich CD8$^+$ T cell populations one week after HIFU. uMAP plots at the 7 day timepoint of (A) control, (B) mechanically ablated, (C) mechanical HIFU pretreated with immunotherapy, and immunotherapy alone tumors. Black and red arrows indicate immune populations that change with treatment. Black arrows indicate Cd8a$^+$ populations, red arrows indicate Cd206$^+$,Cd86$^+$ cell populations, and blue arrows indicate Gr1$^+$, Cd11b$^+$ cell populations. Individual uMAPs were generated using uMAP v3.1 on FlowJo v10.7 (https://www.flowjo.com/exchange/#/plugin/profile?id=6) using all markers except CD45 and Live/Dead as parameters with 15 nearest neighbors and 0.01 minimum distance.

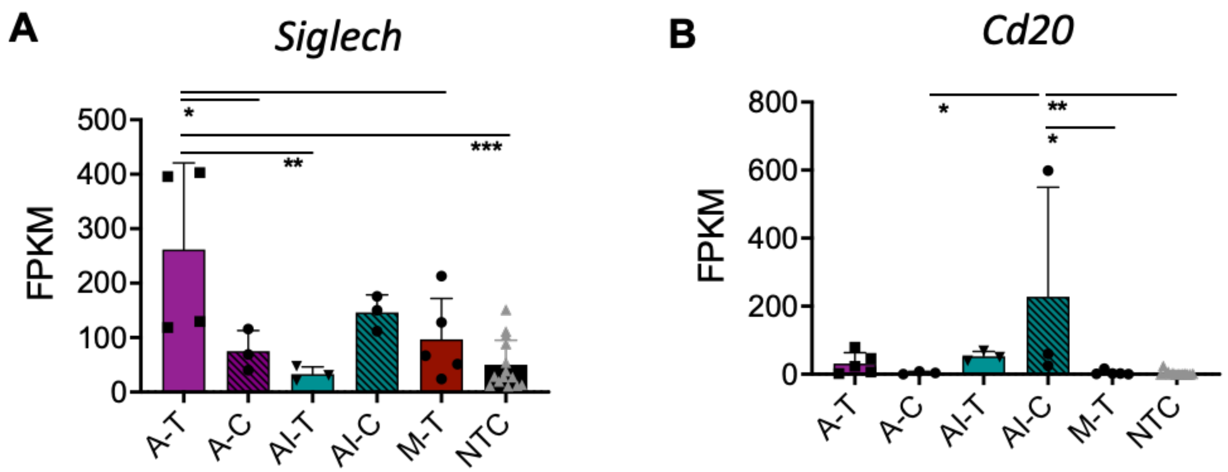

Supplementary Figure S8. Humoral response to HIFU. FPKM values of (A) *Siglech* and (B) *Cd20*.

Supplementary Table S1: Top gene ontology terms for ECM remodeling and wound healing for each treatment

**Ablation+Immunotherapy**			**Thermal Ablation**			**Mechanical ablation**		
GO Term	Genes	P Value	GO Term	Genes	P Value	GO Term	Genes	P Value
GO:0006935 Chemotaxis	46	1.45×10^{-13}	GO:0007155 Cell adhesion	165	5.55×10^{-30}	GO:0006935 Chemotaxis	31	2.37×10^{-11}
GO:0007155 Cell adhesion	106	2.98×10^{-10}	GO:0001525 Angiogenesis	100	3.97×10^{-26}	GO:001525 Angiogenesis	45	8.23×10^{-11}
GO:0045766 Positive regulation of angiogenesis	36	2.71×10^{-7}	GO:0030198 ECM organization	40	2.82×10^{-8}	GO:0007155 Cell adhesion	64	2.55×10^{-8}
GO:0032715 Negative regulation of IL-6 production	16	3.05×10^{-7}	GO:0030199 Collagen fibril organization	21	3.00×10^{-8}	GO:0030199 Collagen fibril organization	14	3.65×10^{-7}
GO:0030574 Collagen catabolic process	14	5.75×10^{-7}	GO:0030574 Collagen catabolic process	16	4.76×10^{-8}	GO:0045766 Positive regulation of angiogenesis	24	1.47×10^{-6}
GO:0001525 Angiogenesis	55	1.57×10^{-6}	GO:0042060 Wound healing	34	1.61×10^{-7}	GO:0007160 Cell-matrix adhesion	18	4.98×10^{-6}
GO:0042060 Wound healing	27	2.15×10^{-5}	GO:0032755 Positive regulation of IL-6 production	22	2.14×10^{-6}	GO:0032755 Positive regulation of IL-6 production	13	6.70×10^{-5}
GO:0032733: Positive regulation of IL-10 production	12	2.06×10^{-5}	GO:0032733: Positive regulation of IL-10 production	9	0.009421	GO:0042060 Wound healing	17	2.20×10^{-4}

Supplementary Table S2: Innate immune response gene ontology terms for each treatment

Ablation+Immunotherapy			Thermal Ablation			Mechanical ablation		
GO Term	Genes	P Value	GO Term	Genes	P Value	GO Term	Genes	P Value
GO:0002376 Immune system process	147	2.87×10^{-41}	GO:0002376 Immune system process	115	2.80×10^{-16}	GO:0002376 Immune system process	55	1.63×10^{-8}
GO:0045087 Innate immune response	111	2.53×10^{-18}	GO:0032496 Response to LPS	70	4.05×10^{-14}	GO:0032496 Response to LPS	32	1.81×10^{-6}
GO:0006955 Immune response	81	2.61×10^{-15}	GO:0006955 Immune response	69	5.62×10^{-7}	GO:0045084 Innate immune response	50	4.93×10^{-6}
GO:0032496 Response to LPS	63	1.27×10^{-13}	GO:0045087 Innate immune response	92	7.40×10^{-7}	GO:0006955 Immune response	30	0.003484
GO:0042832 Response to protozoan	20	3.14×10^{-11}	GO:0014068 Positive regulation of phagocytosis	20	9.20×10^{-6}	GO:0051607 Defense response to virus	21	0.003899

Supplementary Table S3 Enriched adaptive immune response GO terms for each treatment

Ablation+Immunotherapy			Thermal Ablation			Mechanical ablation		
GO Term	Genes	P Value	GO Term	Genes	P Value	GO Term	Genes	P Value
GO:0002250 Adaptive immune response	52	2.16×10^{-14}	GO:0042110 T cell activation	19	7.80×10^{-8}	GO:0046641 Cellular response to IFN gamma	12	0.003102
GO:0042102 Positive regulation of T cell proliferation	30	2.10×10^{-11}	GO:0050870 Positive regulation of T cell activation	12	1.93×10^{-6}	GO:0046641 Positive regulation of alpha-beta T cell proliferation	4	0.02751
GO:0050852 T cell receptor signaling pathway	25	4.26×10^{-10}	GO:0042102 Positive regulation of T cell proliferation	25	2.00×10^{-6}	GO:0042098 T cell proliferation	5	0.05936
GO:0042110 T cell activation	17	3.56×10^{-7}	GO:0030217 T cell differentiation	17	5.70×10^{-5}			
GO:0032729 Positive regulation of IFNγ production	21	7.19×10^{-7}	GO:0002250 Adaptive immune response	33	0.002257			
GO:0031295 T cell costimulation	14	1.07×10^{-6}	GO:0032729 Positive regulation of IFNγ production	16	0.003623			
GO:0050870 Positive regulation of T cell activation	11	4.47×10^{-6}	GO:0031295 T cell costimulation	10	0.004618			

Supplementary Table S4 Cytokine and chemokine enriched GO terms for directly-treated tumors

Ablation+Immunotherapy			Thermal Ablation			Mechanical ablation		
GO Term	Genes	P Value	GO Term	Genes	P Value	GO Term	Genes	P Value
GO:0030593 Neutrophil chemotaxis	27	2.64×10^{-8}	GO:0030593 Neutrophil chemotaxis	33	6.76×10^{-11}	GO:0030593 Neutrophil chemotaxis	20	2.63×10^{-8}
GO:0001816 Cytokine production	13	8.21×10^{-6}	GO:0019221 Chemokine mediated signaling pathway	44	6.98×10^{-7}	GO:0002523 Leukocyte migration involved in inflammatory response	8	4.49×10^{-6}
GO:0070098 Chemokine mediated signaling pathway	20	8.69×10^{-6}	GO:0090023 Positive regulation of neutrophil chemotaxis	16	7.87×10^{-7}	GO:0071347 Cellular response to IL-1	17	2.95×10^{-5}
GO:0032722 Positive regulation of chemokine production	10	2.13×10^{-4}	GO:0050715 Positive regulation of cytokine secretion	17	3.18×10^{-6}	GO:0070098 Chemokine mediated signaling pathway	13	1.20×10^{-4}
GO:0071345 Cellular response to cytokine stimulus	13	2.27×10^{-4}	GO:0070098 Chemokine mediated signaling pathway	22	6.19×10^{-6}	GO:0002544 Chronic inflammatory response	5	4.56×10^{-4}
GO:0050715 Positive regulation of cytokine secretion	13	3.13×10^{-4}	GO:0071347 Cellular response to IL-1	25	1.39×10^{-4}	GO:0019221 Cytokine mediated signaling pathway	21	7.65×10^{-4}

Supplementary Table S5 Enriched B cell GO terms for each treatment

Ablation+Immunotherapy			Thermal Ablation			Mechanical ablation		
GO Term	Genes	P Value	GO Term	Genes	P Value	GO Term	Genes	P Value
GO:0050853 B cell receptor signaling pathway	25	2.88×10^{-9}	GO:0050861 Positive regulation of B cell receptor signaling pathway	6	8.32×10^{-4}	GO:0030889 Negative regulation of B cell proliferation	7	5.68×10^{-4}
GO:0045579 Positive regulation of B cell differentiation	11	8.99×10^{-6}	GO:0030890 Positive regulation of B cell proliferation	15	0.001384	GO:0001782 B cell homoeostasis	7	0.004588
GO:0042113 B cell activation	12	2.69×10^{-4}	GO:0050853 B cell receptor signaling pathway	15	0.01553	GO:0002322 B cell proliferation involved in immune response	3	0.03438
